# Targeted imaging of uPAR expression in vivo with cyclic AE105 variants

**DOI:** 10.1038/s41598-023-43934-w

**Published:** 2023-10-11

**Authors:** Julie Maja Leth, Estella Anne Newcombe, Anne Louise Grønnemose, Jesper Tranekjær Jørgensen, Katrine Qvist, Anne Skovsbo Clausen, Line Bruhn Schneider Knudsen, Andreas Kjaer, Birthe Brandt Kragelund, Thomas Jørgen Dyreborg Jørgensen, Michael Ploug

**Affiliations:** 1 Finsen Laboratory, Copenhagen University Hospital – Rigshospitalet, 2200 Copenhagen N, Denmark; 2 Biotech Research and Innovation Centre (BRIC), University of Copenhagen, 2200 Copenhagen N, Denmark; 3 Structural Biology and NMR Laboratory, Copenhagen N, Denmark; 4 REPIN, Copenhagen N, Denmark; 5 The Linderstrøm Lang Centre for Protein Science, Department of Biology, University of Copenhagen, Ole Maaloes Vej 5, 2200 Copenhagen N, Denmark; 6 Department of Biochemistry and Molecular Biology, University of Southern Denmark, 5230 Odense M, Denmark; 7 Department of Clinical Physiology and Nuclear Medicine and Cluster for Molecular Imaging, Copenhagen University Hospital – Rigshospitalet, Copenhagen N, Denmark; 8 Department of Biomedical Sciences, University of Copenhagen, Copenhagen N, Denmark

**Keywords:** Biochemistry, Biophysics, Cancer, Structural biology, Biomarkers, Oncology

## Abstract

A comprehensive literature reports on the correlation between elevated levels of urokinase-type plasminogen activator receptor (uPAR) and the severity of diseases with chronic inflammation including solid cancers. Molecular imaging is widely used as a non-invasive method to locate disease dissemination via full body scans and to stratify patients for targeted treatment. To date, the only imaging probe targeting uPAR that has reached clinical phase-II testing relies on a high-affinity 9-mer peptide (AE105), and several studies by positron emission tomography (PET) scanning or near-infra red (NIR) fluorescence imaging have validated its utility and specificity in vivo. While our previous studies focused on applying various reporter groups, the current study aims to improve uPAR-targeting properties of AE105. We successfully stabilized the small uPAR-targeting core of AE105 by constraining its conformational landscape by disulfide-mediated cyclization. Importantly, this modification mitigated the penalty on uPAR-affinity typically observed after conjugation to macrocyclic chelators. Cyclization did not impair tumor targeting efficiency of AE105 in vivo as assessed by PET imaging and a trend towards increased tracer uptake was observed. In future studies, we predict that this knowledge will aid development of new fluorescent AE105 derivatives with a view to optical imaging of uPAR to assist precision guided cancer surgery.

## Introduction

The urokinase-type plasminogen activator receptor (uPAR) is a glycolipid-anchored membrane protein belonging to the Ly-6/uPAR (LU) protein domain family^1,2^. In a physiological context, uPAR plays a dual role in fibrin surveillance and in cell adhesion and migration, respectively. First, uPAR focalizes plasminogen activation onto cell surfaces through its high-affinity binding to the urokinase-type plasminogen activator (uPA) and the resultant generation of plasmin acts to resolve spontaneous fibrin depositions, thus mitigating subsequent chronic inflammation^3^. Second, uPAR facilitates cell adhesion by its low affinity binding to the matrix-embedded form of vitronectin^4–7^. Several observational studies correlate elevated uPAR levels with the severity and progression of a number of diseases supporting chronic inflammation^8^ such as a plethora of solid cancers^9–11^, kidney disease^12^, rheumatoid arthritis^13,14^, HIV infection^15^, COVID-19 infection^16^, and atherosclerosis^17^. Whether uPAR function has any causality per se in these disease associations remains in most cases unclear or controversial and there is little genetic or epidemiological evidence to support such causality. Notably, uPAR-deficient mice are viable with only very mild overt phenotypes, but these unchallenged mice do have late-onsets of *e.g.* chronic hepatic inflammation—a condition observed in aged uPAR^−/−^ mice with unresolved fibrin deposition^3,18,19^.

The lack of severe overt phenotypes in uPAR-deficient mice combined with low uPAR-expression levels in homeostatic and non-inflamed tissues, prompted a change in strategies for in vivo uPAR-targeting from being primarily focused on function-inhibition approaches^20,21^ to rely more on targeted-cytotoxic approaches to eradicate uPAR-expressing cells^22–29^. Parallel to those new attempts to design cytotoxic uPAR-targeted therapies, others developed several non-invasive imaging approaches to visualize uPAR expression in vivo—thus completing a possible theranostic pipeline for uPAR in clinical oncology^30–36^. The virtue of these uPAR-specific imaging platforms is that they (i) can aid patient stratification, (ii) can follow treatment responses using positron emission tomography (PET) scanning^31,37–40^, and (iii) can potentially be used to increase cancer surgery precision by fluorescence-guided intraoperative imaging^33,34,41,42^. Furthermore, the elevated expression of uPAR in chronically inflamed tumor-stroma microenvironments makes it an ideal candidate for fluorescence-guided intraoperative imaging during cancer resection^30^.

Due to the conformational flexibility of its large hydrophobic uPA-binding cavity, uPAR-targeting with small molecules is challenging^4,43–48^. Despite huge efforts in developing small antagonists targeting this particular ligand-binding site^27,47,49–52^ none have so far, to the best of our knowledge, reached clinical testing—except in the setting of non-invasive cancer imaging where variants of a small peptide AE105 have been applied to patients and some of these probes are currently in phase-1 or phase-2 clinical trials^30,31,37,38,42^. This small uPAR-targeting peptide was originally developed by affinity-maturation using a 15-mer antagonist peptide, discovered by phage-display, as a template^53^, and our resultant lead compound (AE105) is a 9-mer peptide containing a mixture of L-, D-, and non-natural amino acids^54^. The core of AE105 folds into a short amphipathic α-helix on binding to the flexible uPA-binding cavity in uPAR and this tight interaction (K_D_ ~ 4 nM) traps uPAR in a semi-open conformation^4,45,55–57^. Importantly, the X-ray structure of uPAR in complex with a derivative of AE105^57^ shows that there is ample free space at the N-terminus suggesting that modifications with various macrocyclic chelators for PET-imaging^32,58–60^ or near-infrared fluorescent probes for optical imaging^33,61,62^ can theoretically be accommodated at this site without paying a detrimental penalty on affinity and specificity. Guided by structural considerations, we have in the present study primarily focused on optimizing the uPAR-binding core of AE105 by introducing a disulfide bond in its C-terminal helix binding core. Furthermore, we explored modifications at the termini of AE105, including an N-terminal linker, a macrocyclic chelator, and neutralizing the negatively charged C-terminal by amidation.

## Results

### Stabilizing the uPAR-binding core of AE105 by disulfide cyclization

**Figure 1:**
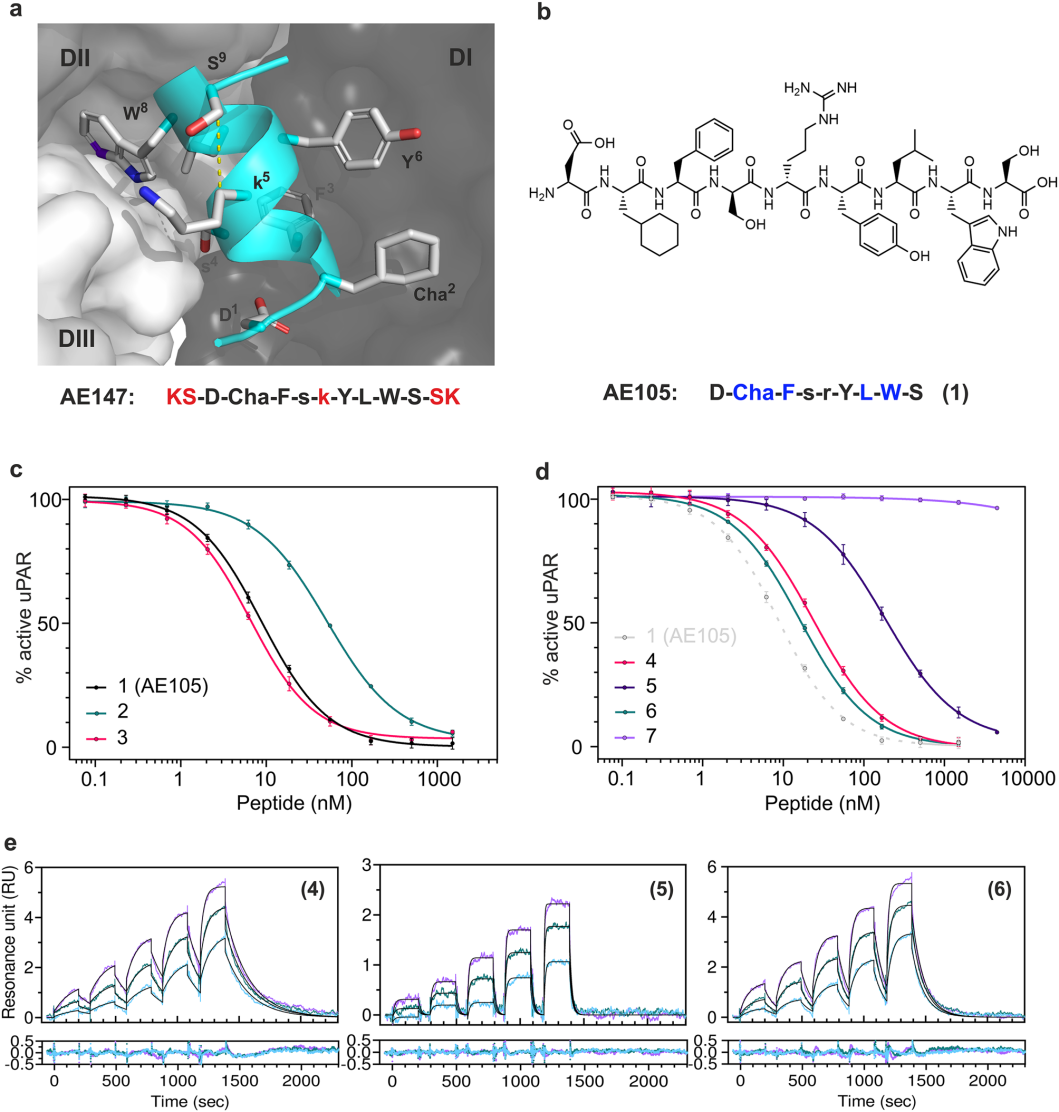
Introduction of a disulfide into the AE105 core. (**a**) Crystal structure of AE147 bound in the uPA-binding cavity of uPAR^57^. This cartoon representation shows the helical AE105 core with side chains as stick. The α-helix is formed by the residues Asp^1^ to Ser^10^. The individual LU-domains in uPAR are shown in a surface representation; DI (*dark gray*), DII (*gray*), DIII (*light gray*). The C_β_-distance between D-Lys^5^ and L-Ser^9^ is highlighted by the yellow dotted line (4 Å). (**b**) Chemical structure of AE105 (compound **1**). (**c**) Competition of the uPA•uPAR interaction by serial threefold dilutions of AE105 **(1)**, AE105 D-Lys^5^ **(2)**, and AE105 with a [D-Cys^5^;L-Cys^9^]–disulfide bond **(3)**. (**d**) Same analyses with an N-terminal EEGsGG spacer **(4)**, with D-Ser^5^ **(5)**, with D-Cys^5^ and L-Cys^9^ **(6)**, and with D-Cys^5^, L-Pro^6^ and L-Cys^9^ **(7)**. (**e**) Real-time binding kinetics of the interaction between immobilized uPAR and antagonistic peptides using three rounds of single-cycle injections as measured by SPR. Left figure shows sensorgram for **(4)**, middle figure for **(5)**, and right figure the corresponding peptide with the [D-Cys^5^;L-Cys^9^]–disulfide **(6)**. Concentrations used for **(4)** and **(6)** were: 1.6–25 nM; 3–50 nM; and 6–100 nM, whereas **(5)** was analyzed at higher concentration due to its lower affinity (6.25–100 nM; 12.5–200 nM; and 25–400 nM). Non-linear global fitting is shown as a thin black line and residuals are shown in the graph below.

From our previous biophysical studies with hydrogen–deuterium exchange mass spectrometry (HDX-MS), we know that the 9-mer antagonist peptide AE105 is disordered in aqueous solutions, but we also know from our studies with X-ray crystallography that the core of AE105 folds upon uPAR-binding into a short α-helix with little flexibility in the bound state^55,57^, similar to other disordered peptides^63^. The α-helical structure of the core of the uPAR-bound AE105 **(1)** is illustrated in Fig. 1a. The AE105 derivatives used in this study are numbered consecutively as they appear in the text (in bold). Their sequences and binding properties are summarized in Table 1.

**Table 1:** uPAR binding properties of AE105-derivatives.

	Spacer	Sequence	C-term	IC_50_ (nM)	K_D_ (nM)	*k*_*on*_ (10^5^ M^−1^ s^−1^)	*k*_*off*_ (10^–3^ s^−1^)	*T*_*m*_ (°C)
1		DChaFsrYLWS	OH	8.8 ± 0.2	2.52	15.68 ± 0.05	3.96 ± 0.01	64.8 ± 0.4
2		DChaFs**k**YLWS	OH	50.9 ± 2.0	50.2	5.94 ± 0.03	29.8 ± 0.1	–
3		DChaFs**c**YLW**C**	OH	6.5 ± 0.3	3.32	10.98 ± 0.04	3.65 ± 0.01	~ 70
4	EEGsGG	DChaFsrYLWS	OH	23.6 ± 0.9	19.9	2.80 ± 0.01	5.58 ± 0.01	55.2 ± 0.2
5	EEGsGG	DChaFs**s**YLWS	OH	184 ± 13	268	2.01 ± 0.02	54.0 ± 0.3	–
6	EEGsGG	DChaFs**c**YLW**C**	OH	16.3 ± 0.3	16.9	6.11 ± 0.03	10.33 ± 0.03	60.6 ± 0.7
7	EEGsGG	DChaFs**cP**LW**C**	OH	> 1.5 × 10^3^	N.B	N.B	N.B	–
8		DChaFsrYLWS	NH_2_	2.1 ± 0.1	0.53	18.90 ± 0.03	0.998 ± 0.001	≥ 70
9		DChaFs**c**YLW**C**	NH_2_	8.9 ± 0.3	2.90	7.12 ± 0.02	2.063 ± 0.003	≥ 70
10	EEGsGG	DChaFsrYLWS	NH_2_	4.2 ± 0.2	2.52	5.95 ± 0.01	1.497 ± 0.002	~ 65
11	EEGsGG	DChaFs**c**YLW**C**	NH2	15 ± 1	10.0	5.00 ± 0.02	5.015 ± 0.008	~ 65
DOTA-conjugated peptides								
1b		DChaFsrYLWS	OH	**48** ± 2	26.9	2.07 ± 0.01	5.57 ± 0.01	58.8
3b		DChaFs**c**YLW**C**	OH	10.9 ± 0.3	12.5	3.52 ± 0.01	4.40 ± 0.01	~ 70
4b	EEGsGG	DChaFsrYLWS	OH	32.2 ± 0.9	33.6	2.13 ± 0.004	7.17 ± 0.01	55.6 ± 0.03
6b	EEGsGG	DChaFs**c**YLW**C**	OH	15.8 ± 0.4	20.3	4.81 ± 0.01	9.77 ± 0.01	61.3 ± 0.4
8b		DChaFsrYLWS	NH_2_	**8.2** ± 0.1	4.22	4.90 ± 0.01	2.064 ± 0.002	~ 70
9b		DChaFs**c**YLW**C**	NH_2_	7.3 ± 0.2	4.26	6.44 ± 0.02	2.744 ± 0.004	≥ 70
10b	EEGsGG	DChaFsrYLWS	NH_2_	4.8 ± 0.2	3.18	4.297 ± 0.004	1.367 ± 0.001	66.2 ± 0.3
11b	EEGsGG	DChaFs**c**YLW**C**	NH_2_	7.7 ± 0.2	4.97	7.17 ± 0.01	3.562 ± 0.003	~ 70

The IC_50_-values of AE105 derivatives on the uPAR•uPA interaction were obtained by SPR measurements. The IC_50_-values were determined by fitting to a four-parameter dose–response model (n = 3). Standard errors (shown as ±) are derived from the global fitting procedure. SPR real time binding kinetics analyses with single cycle protocols provided association (*k*_on_) and dissociation (*k*_off_) rate constants for the interactions between peptides in solution and immobilised uPAR. Fitting with non-linear regression to a simple bimolecular interaction model yielded the kinetic rate constants and the K_D_. Standard deviations refer to parameters derived directly from the fitting procedures. Apparent melting temperatures (*T*_*m*_) were determined by nano-DSF and calculated as the first derivative of the fluorescence ratio (350 nm/330 nm); n = 3. Letters in **bold**: Residues that are changed compared to the AE105 sequence. N.B; No binding, (–); not measured. Numbers in **bold**: IC_50_-values that are > twofold higher when DOTA is conjugated to the peptide compared to without.

It should be emphasized that this X-ray structure was not solved for uPAR complexed to AE105, but for a complex with an AE105 derivative having a higher solubility (AE147). Differences between AE147 and AE105 are marked in red in Fig. 1a. As a first attempt to improve the uPAR-targeting core of AE105, we explored the possibility of introducing a disulfide bond at positions *i* and *i* + 4 to increase the helix propensity of the core of AE105 with the aim of reducing the entropic penalty on uPAR binding and increasing its binding affinity. Inspection of the crystal structure for uPAR•AE147 highlights a single position suitable for the introduction of a disulfide bond in the α-helical core, namely between D-Lys^5^ and L-Ser^9^ with an optimal C_β_-C_β_ distance of 4.0 Å (Fig. 1a). As the sequence of AE105 and AE147 differ at position 5 (Fig. 1a,b), we first measured the ability of AE105 with D-Arg^5^ **(1)** as well as a derivative of AE105 with D-Lys^5^ **(2)** to compete with the high-affinity uPA•uPAR interaction (*K*_*D*_ of 20 pM^48^). This was done using an in-solution competition setup with surface plasmon resonance (SPR) as detection^4^. The conservative replacement of D-Arg^5^ in AE105 with D-Lys^5^ **(2)** led to a 5.8-fold *increase* in the IC_50_-value for competing the uPA•uPAR interaction—raising it from 8.8 nM to 51 nM (Fig. 1c). Despite this reduced potency, the introduction of a disulfide at [D-Cys^5^;L-Cys^9^] in AE105 **(3)** led to a 1.4-fold *decrease* in the IC_50_-value—lowering it to 6.5 nM (Fig. 1c), thus clearly showing the beneficial effects of this disulfide bond. Since AE105 has limited solubility, we performed additional studies with peptides having a hydrophilic spacer at the N-terminus (Fig. 1d). Adding this spacer to AE105 **(4)** led to a 2.7-fold increase in IC_50_. When D-Arg^5^ was replaced with D-Ser^5^ as a surrogate for D-Cys^5^ in this context **(5)**, we measured an IC_50_ of 184 nM corresponding to a 7.8-fold decrease in potency compared to **(4)**. When the [D-Cys^5^;L-Cys^9^]–disulfide was introduced into this framework **(6)**, we observed a substantial decrease in the IC_50_-value (16.3 nM), which corresponds to an increase in potency of 11-fold compared to **(5)** and 1.4-fold compared to **(4)**. The inhibitory potency of this cyclized peptide was ablated by replacing L-Tyr^6^ in **(6)** with the helix-breaking L-Pro^6^ **(7)**.

As an orthogonal method, we measured the direct real-time binding kinetics between immobilized uPAR and the antagonistic peptides, also by SPR. The SPR sensorgrams for three extended versions of AE105 are shown in Fig. 1e; one with the original D-Arg^5^ **(4)**; one with D-Ser^5^ **(5)**; and one with the [D-Cys^5^;L-Cys^9^]–disulfide bond **(6)**. Global fitting of the combined data from three rounds of single-cycle injections to a simple 1:1 binding model proved robust for all three peptides (Fig. 1e). The derived equilibrium dissociation constants (*K*_*D*_) for **(4)** and **(6)** were comparable (19.9 nM and 16.9 nM, respectively), whereas **(5)** had a considerably reduced affinity with a *K*_*D*_ of 268 nM. The kinetics of the three peptides align well with the IC_50_-values determined in competition experiments (Fig. 1d). Notwithstanding that **(4)** and **(6)** have similar *K*_*D*_ values, their kinetic rate constants differ. The association rate constant (*k*_*on*_) for the [D-Cys^5^;L-Cys^9^] peptide **(6)** was improved ~ twofold compared to **(4)**, while the dissociation rate constant (*k*_*off*_) in contrast suffered a ~ twofold impairment giving rise to a similar* K*_*D*_ for the interaction with uPAR (Table 1).

Different combinations in the chirality of cysteines forming the [D-Cys^5^;L-Cys^9^]–disulfide bond were also explored, but none was as efficient as the parent [D-Cys^5^;L-Cys^9^] peptide **(6)**, as illustrated in Fig S1a. We also tested less obvious positions for disulfide introduction in AE105 and as expected these were all inferior to the [D-Cys^5^;L-Cys^9^] peptide **(6)** in competing the uPA•uPAR interaction (Figure S1a).

### Amidation of the C-terminus in AE105

**Figure 2:**
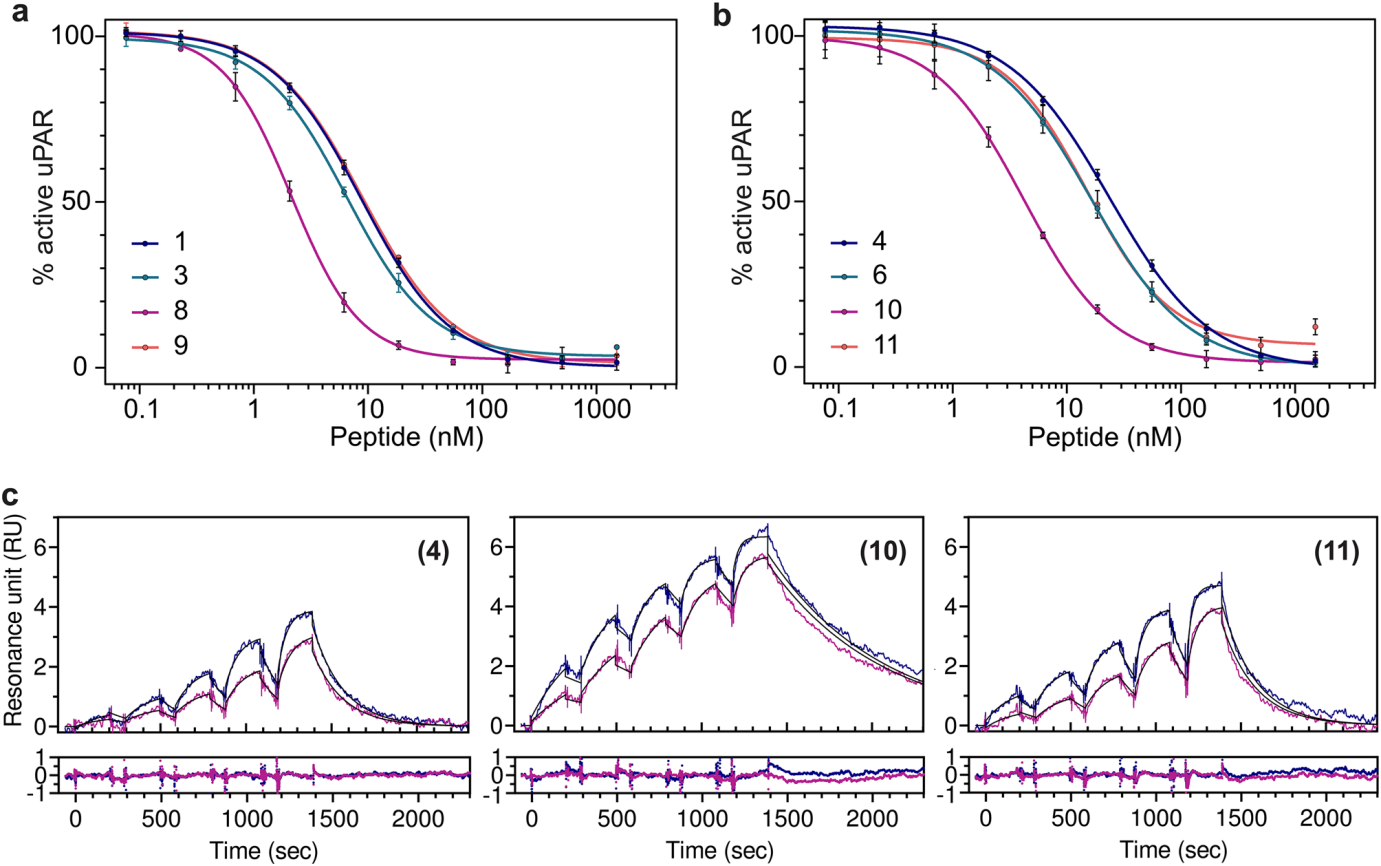
Amidation of C-terminal Ser^9^ in the AE105 core. (**a**) Competition of the uPA•uPAR interaction by serial threefold dilutions of AE105 without **(1)** and with amidation **(8)**, and AE105 with [D-Cys^5^;L-Cys^9^]–disulfide without **(3)** and with amidation **(9)**. (**b**) Same analyses of AE105 with an N-terminal EEGsGG linker **(4)** and with amidation **(10)**, and AE105 with [D-Cys^5^;L-Cys^9^]–disulfide and an N-terminal EEGsGG linker **(6)** and with amidation **(11)**. (c) Real-time binding kinetics for the interaction between immobilized uPAR and peptides using two rounds of single-cycle injections as monitored by SPR. Left figure shows AE105 with linker **(4)**, middle figure shows AE105 with linker and amidation **(10)**, and right figure shows AE105 with the [D-Cys^5^;L-Cys^9^]–disulfide, the EEGsGG linker, and amidation **(11)**. Concentrations of peptides were: 1.6–25 nM and 3–50 nM. Non-linear global fitting to a 1:1 model is shown as a thin black.

The parent 15-mer antagonist peptide originally selected by phage-display had a C-terminal extension compared to AE105^53,64^. That truncation introduced a C-terminal carboxylate in AE105 at Ser^9^, which was not present in the original 15-mer phage-display peptide as Ser^9^ here was engaged in peptide bonding to residue 10. Introducing a negatively charged carboxylate at the C-terminus of an α-helix may however destabilize the inherent helix propensity by perturbations of the helix dipole moment^65–67^. Based on these considerations, we tested the impact of amidating the carboxylate of Ser^9^ in AE105 **(1)** and its derivatives **(3)**,** (4)**, and **(6)**. When we assessed the ability of the amidated peptides to compete with the uPA•uPAR interaction, we found that amidating the linear peptides **(1)** and **(4)** led to a 4.2- and a 5.6-fold increase in their potency resulting in IC_50_-values of 2.1 nM **(8)** and 4.2 nM **(10)**. A similar beneficial effect of C-terminal amidation was however not observed for the disulfide bonded peptides *i.e.*, **(3)** *vs.* **(9)** and **(6)** *vs.* **(11)**, as illustrated in Fig. 2a,b and Table 1. Similar impacts were observed for the kinetics of the peptide interactions with immobilized uPAR (Fig. 2c). The beneficial effects on affinity were mainly ascribed to a slower *k*_*off*_ for the amidated peptides (Table 1).

### Evidence for an increased helix propensity after disulfide cyclization: NMR and HDX-MS

**Figure 3:**
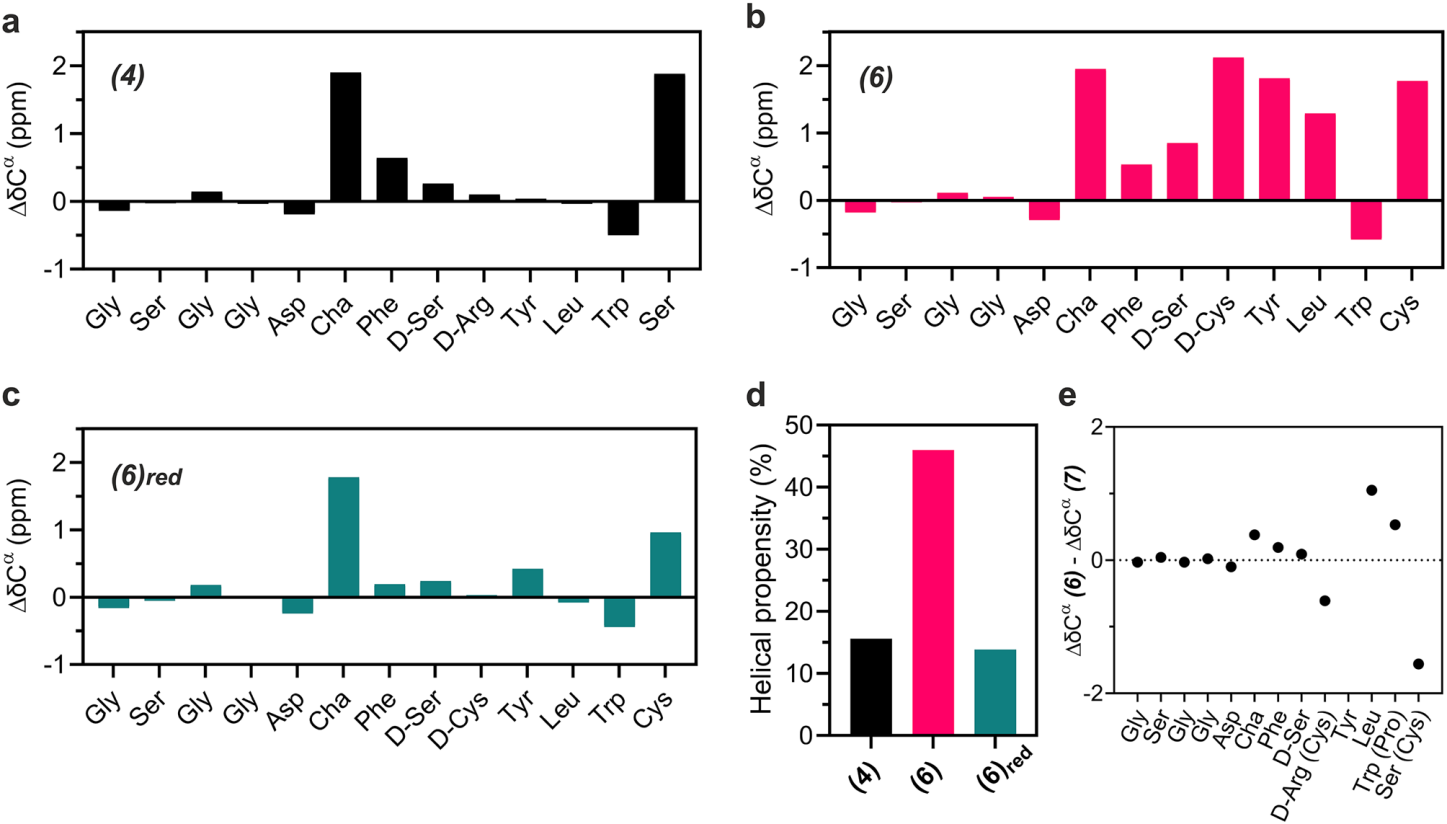
Impact of disulfide on secondary structure by NMR secondary chemical shifts (SCSs) of C^α^. (**a**) shows the C^α^ SCSs for the extended version of AE105 **(4),** which in general are weak except for Cha^2^ and Ser^9^. (**b**) The corresponding values after introducing the [D-Cys^5^;L-Cys^9^]–disulfide bond **(6).** Note the increased C^α^ SCSs values in the C-terminal region (except for Trp^8^), which indicates a gain in helicity of this region. (**c**) shows that this gain in helicity is lost upon reduction of this disulfide bond in peptide **(6)**. (**d**) compares the helical propensity from Cha^2^-Leu^7^ in **(4)** and **(6)** using 3.1 ppm as SCS-reference for 100% helicity^68^. (**e**) Differences in C^α^ SCSs of **(6)** and **(7)**.

**Figure 4:**
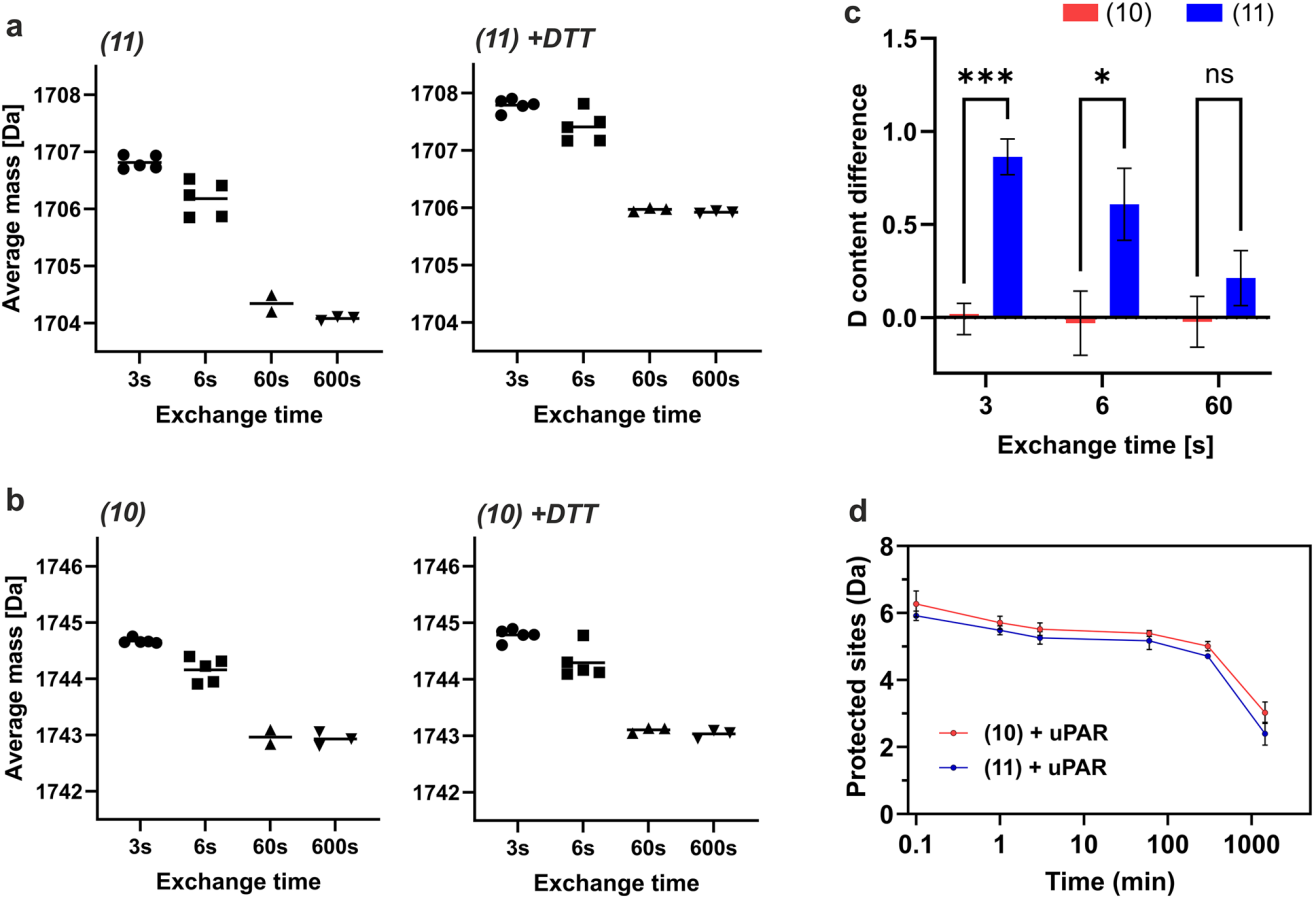
Impact of disulfide on structural protection measured by HDX-MS. (**a**) and (**b**) show the average masses of peptides **(11)** and **(10)** after various periods of D-to-H exchange in the absence (left) or presence (right) of DTT at pH 6.3, 0 °C as measured by HDX-MS. Experiments were repeated 3–6 times: n = 5 for 3 s and 6 s, and n = 3 for 60 s and 600 s. (**c**) Disulfide bond formation in **(11)** induces protection of its backbone amides. The difference in deuterium content between samples in the absence and presence of DTT are shown for **(11)** (blue bars) and **(10)** (red bars) after D-to-H exchange for 3, 6, and 60 s at pH 6.3, 0 °C as measured by HDX-MS. (**d**) Level of uPAR-induced protection of H-to-D exchange in **(10)** and **(11)** was calculated as the difference in deuterium content between maximally labeled peptides and peptides bound to uPAR, (H-to-D exchange experiments were performed triplicates at pH_read_ 7.4, at room temperature).

To obtain direct evidence showing that introduction of the [D-Cys^5^;L-Cys^9^]–disulfide bond increases the inherent helix propensity of AE105, we employed nuclear magnetic resonance (NMR) spectroscopy (Fig. 3) and HDX-MS (Fig. 4) for the analyses of unbound peptide ligands.

We first assigned the chemical shifts of the peptides using natural abundance detected 2D NMR spectra **(**Figs S2 and S3). With analysis of the secondary chemical shifts (SCSs) of C^α^, which are direct measures of φ,ψ angles in the backbone, we showed that the helical propensity was notably higher in the presence of the [D-Cys^5^;L-Cys^9^]–disulfide bond **(6)** compared to the corresponding linear peptide **(4)** *i.e.,* 15% *vs.* 45% (Fig. 3d). From the SCSs it was evident that the gain in helicity originates predominantly from residues positioned next to and between the cysteines forming the disulfide bond (Fig. 3a,b). To validate that this gain in helicity was solely caused by the formation of the disulfide bond and not by alterations in the sequence composition, we performed a parallel analysis of **(6)** following reduction of its disulfide bond. Breaking the disulfide bond by reduction lowered the C^α^ SCSs to those of the parent peptide **(4)** confirming that the increased helix propensity of **(6)** is attributed to the disulfide bond formation (Fig.  3c,d). Along the same line of evidence, substituting L-Tyr^6^ with the helix-breaking amino acid proline **(7)** reduced the helix propensity of **(6)** and at the same time led to structural heterogeneity within the peptide, likely caused by *cis–trans* proline isomerization. Although the C^α^ SCSs are high for the two cysteines, and thus can indicate some helicity of **(7)**, the overall structure of the binding core is not identical to **(6)**, and in particular the orientation of the Leu and Trp are distorted (Fig. 3e)—both residues are hotspots for uPAR binding^54,64^.

We also noted that **(5)**, in which the D-Arg^5^ is replaced with D-Ser^5^ as a surrogate for D-Cys^5^, as well as the reduced state of **(6)**, both exhibited a reduced overall helix propensity compared to **(4)**. A possible explanation for this effect comes from the likely formation of a salt bridge between L-Asp^1^ and D-Arg^5^ in **(4)**, which is known to stabilize helical peptides^69^. The presence of such a stabilizing interaction may also explain the increase in IC_50_ on substituting Arg^5^ with Lys^5^, as arginine forms stronger salt bridges than lysine and thus has a stronger helix stabilizing effect^69^.

We next proceeded to use HDX-MS as an orthogonal method to assess if we could demonstrate an increase in helical propensity. For these experiments, we wanted to measure the helical propensity for the amidated versions of **(4)** and **(6)**, *i.e.*, **(10)** and **(11)**, respectively. We used *D-to-H exchange* to minimize deuterium retention in the sidechain of D-Arg^5^—a factor that may confound studies using H-to-D exchange due to the relatively slow exchange rate of guanidino hydrogens at quench conditions^55,70^.

If the disulfide bond in **(11)** were to increase α-helical propensity, then reinforced hydrogen-bonding would protect the backbone amides from solvent exchange. Such protection can be probed by D-to-H exchange of backbone amide groups, which should proceed slower in the absence of DTT (*i.e.*, with an intact disulfide bond) than in the presence of DTT (*i.e.*, with a cleaved disulfide bond). To determine if such a difference exists in the exchange kinetics for **(11)**, we measured the difference in the deuterium content of **(11)** in the absence and presence of DTT (Fig. 4a,c). A significant difference in the deuterium content for **(11)** was indeed observed at the shortest exchange times (Fig. 4c), providing direct evidence for a disulfide bond-induced protection of its backbone amides. That difference almost disappeared at 60 s reflecting that disulfide bond formation did not induce strong protection against exchange with the solvent. Importantly, the linear peptide **(10)** did not exhibit any difference in its deuterium content demonstrating that the backbone amide hydrogen exchange rates for a peptide without a disulfide bond are identical in the presence and absence of DTT (Fig. 4b,c). The presence of a disulfide bond in **(11)** caused a 2 Da mass shift in average mass for the completely exchanged peptide at 600 s (Fig. 4a). While it is not possible to predict the exchange kinetics of unstructured **(10)** and **(11)** due to the presence of non-canonical amino acids (D-amino acids & cyclohexyl alanine), it is nevertheless important to assess the effect of a disulfide bond on the intrinsic exchange rate. Therefore, we predicted the exchange rate of unstructured pseudo-**(10)** and pseudo-**(11)** based on intrinsic exchange rate constants provided by^70^, where D-amino acids were replaced by L-amino acids, and Cha replaced by Phe (Fig S4). Disulfide bond formation has a negligible effect on the predicted exchange profiles for unstructured peptides (Fig S4) providing further support for a disulfide-bond induced α-helical protection.

To evaluate the structural impact of the [D-Cys^5^;L-Cys^9^]–disulfide on the peptide bound to uPAR, we next probed the deuterium uptake for **(10)** and **(11)** in complex with uPAR by *H-to-D exchange* experiments (Fig. 4d). The level of protection showed that both peptides, independent of the disulfide bond, are highly protected against isotopic exchange in the complex. Even after 24 h of continuous labelling, several sites retain their backbone amide hydrogens (^1^H), reflecting a very tight interaction with uPAR. In the present study, all mass spectra displayed unimodal isotope distributions reflecting that the probability of correlated exchange upon complex dissociation at physiological pH is so low that bimodality is not observed^55^. The level of protection for the linear **(10)** and disulfide bonded **(11)** are nearly identical.

### Thermal stability of uPAR-peptide complexes: NanoDSF

**Figure 5:**
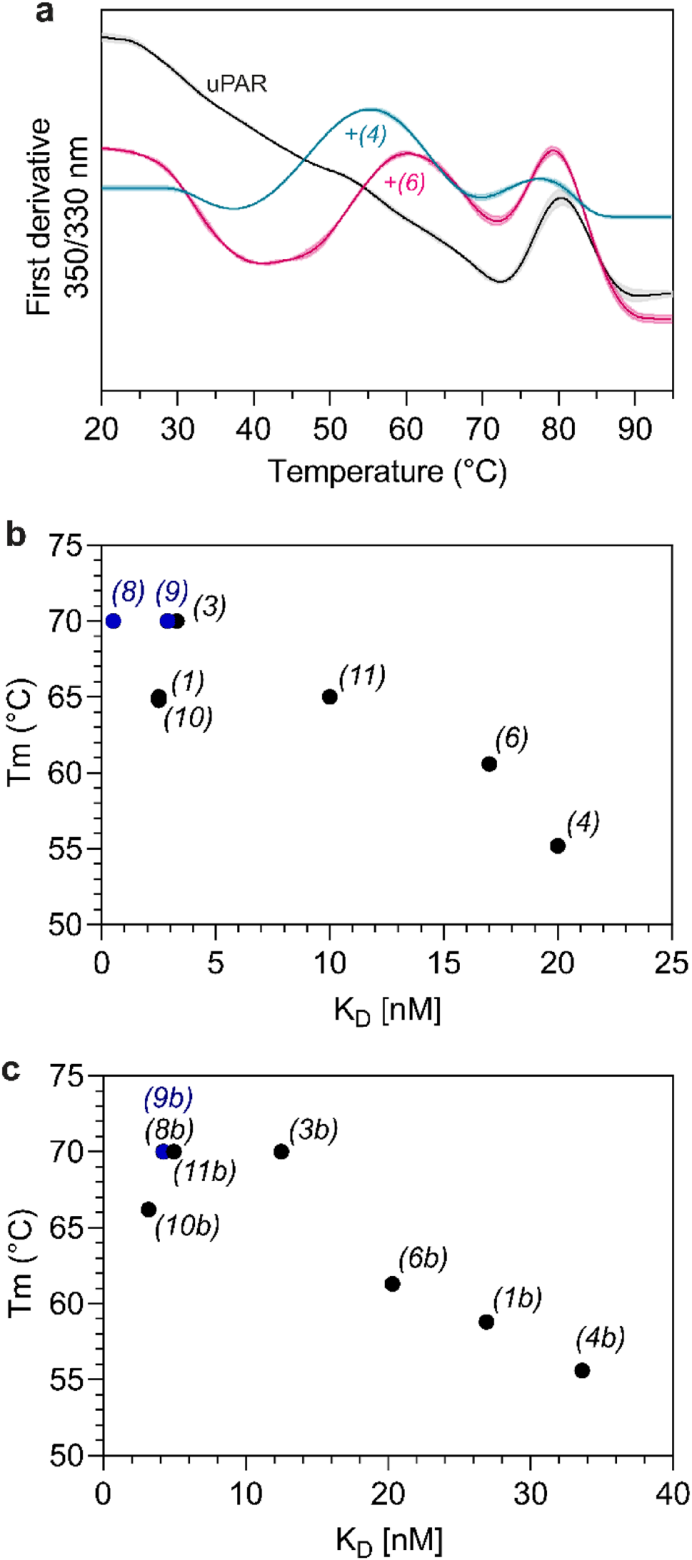
uPAR•peptide complex stability measured by Nano-DSF. (**a**) Unfolding curves of uPAR (black), uPAR in complex with the linear peptide **(4)** (*blue*) or the disulfide peptide **(6)** (*pink*). Curves show the first derivative of the fluorescence ratio (350 nm/330 nm) in a temperature gradient of 20–95 °C. uPAR has an apparent *T*_*m*_ of 72 °C. Binding to **(4)** and **(6)** induce an extra *T*_*m*_ at 55.2 °C and 60.6 °C, respectively (n = 3). Of note, peptides tested alone did not displayed any *T*_*m*_ (not shown). (**b**) Correlation between *K*_*D*_ and apparent *T*_*m*_ for peptides listed in Table 1. Two peptides have *T*_*m*_ ≥ 70 (*blue dots*), and could not be determined reliably due to overlap with the *T*_*m*_ of uPAR*.* (**c**) Correlation between *K*_*D*_ and *T*_*m*_ for DOTA-conjugated peptides listed in Table 1. One peptide with an *T*_*m*_ ≥ 70 is represented by a blue dot.

We next examined how disulfide cyclization, amidation, and spacer addition affect the thermal stability of uPAR•peptide complexes using nano-differential scanning fluorimetry (nano-DSF). Unoccupied uPAR displayed an apparent melting temperature (*T*_*m*_) at 72 °C (Fig. 5a). However, the melting profiles of uPAR•peptide complexes displayed an additional melting transition at lower temperatures—occurring between 55–70 °C (Fig. 5a and Table 1). We interpret this second transition in apparent *T*_*m*_ to reflect changes in the peptide tryptophan environment when the peptide dissociates from uPAR *i.e.*, reporting on the stability of the complex. Using this platform for comparing apparent *T*_*m*_s of various peptide•uPAR complexes, we found that the thermal stability increased by 5 °C for the [D-Cys^5^;L-Cys^9^]–disulfide-bonded peptide **(6)** compared to the corresponding linear peptide **(4)** (Fig. 5a). An identical impact of the disulfide is evident for the shorter versions **(1)** *vs.* **(3)** (Table 1). The introduction of a spacer sequence in **(4)** and **(6)** decreased the apparent *T*_*m*_ with 10 °C as compared to **(1)** and **(3)**, respectively (55.2 °C *vs.* 64.8 °C and 60.6 °C *vs.* ~ 70 °C (Table 1)). In contrast, C-terminal amidation increased the thermal stability of both disulfide-bonded and linear peptides up to approximately 10 °C (Table 1). Of note, we could not determine the exact apparent *T*_*m*_ for all amidated peptides due to overlapping melting transitions with that of uPAR. Nonetheless, we found that disulfide cyclization as well as amidation increased the stability of the uPAR•peptide complex compared to the corresponding control peptides—a relationship emerging for folding and binding complexes^71^. Overall, we find an inverse correlation between apparent *T*_*m*_ and *K*_*D*_—higher apparent *T*_*m*_-values correlated to lower *K*_*D*_s (Fig. 5b).

### Introducing a macrocyclic chelator (DOTA) in AE105: Impact on uPAR binding and stability

So far, we have compared binding and structural properties of AE105 derivatives without the reporter probe required for in vivo imaging. Next, we therefore tested the impact of adding a macrocyclic chelator (DOTA) to the N-terminus of AE105 derivatives. As the uPAR targeting core of AE105 is relatively small (9 residues), the introduction of large reporter groups (macrocyclic chelators or NIR fluorophores) can potentially have deleterious effects on target specificity and efficacy. To address this concern, we probed *i*) the uPAR-binding properties of DOTA-conjugated peptides by SPR and *ii*) the thermal stability of the DOTA-peptide•uPAR complexes by nano-DSF (Table 1). Unexpectedly, we found that DOTA affected the disulfide peptides and the linear peptides differently. For the two short versions of AE105, **(1b)** and **(8b)**, DOTA-conjugation markedly reduced their potency. The IC_50_ values were four to fivefold higher compared to that of the corresponding unconjugated peptides (*i.e.*, **(1)** *vs.* **(1b)** and **(8)** *vs.* **(8b)**, [Table 1]). Of note, peptide **(8)** was the most potent of the unconjugated peptides. This negative impact of DOTA-conjugation was supported by real-time binding kinetics in which the affinity for **(1b)** and **(8b)** was reduced 8–11 fold (Table 1). This reduction was mainly caused by lower association rate constants (*k*_*on*_). Adding a 6-amino acid hydrophilic spacer to the linear peptides in **(4b)** and **(10b)** eliminated the negative impact of DOTA [**(4)** *vs.* **(4b)** and **(10)** *vs.* **(10b)**, (Table 1)].

In contrast to the linear peptides, DOTA-conjugation did not impose the same negative affect on the disulfide-bonded peptides – regardless of whether a spacer was added or not (*i.e.*, peptides **(3)**,** (6)**, (**9)**, **(11)** *vs.* **(3b)**,** (6b)**, (**9b)**, **(11b)**). In fact, the potency of **(11b)** was twofold higher compared to the corresponding unconjugated **(11)** [IC_50_ of 7.7 nM *vs.* 15 nM, (Table 1)]. That relationship was recapitulated by real-time binding kinetics of **(11b)** [K_D_ of 5 nM **(11b)** and K_D_ of 10 nM **(11)**]_._ Thus, the interlocking of the AE105 core in a helical conformation may prevent undesirable long-range interactions with DOTA. Reducing this negative impact from the reporter probe by constraining the helical structure of the uPAR-targeting core of AE105 may be even more relevant in optical imaging where the NIR fluorophore is likely to negatively impact the uPAR targeting efficacy of linear AE105 peptides^62^.

Thermal stability studies of uPAR complexes with DOTA-conjugated peptides by and large recapitulated SPR binding data (Table 1 and Fig. 5c). The negative impact of DOTA-conjugation on the affinity of short AE105 derivatives is thus mirrored by a decrease in apparent *T*_*m*_ for both **(1b)** and **(8b)** in complex with uPAR as compared to the corresponding unconjugated peptides **(1)** and **(8)**. Importantly, we found that DOTA conjugation did not negatively impact peptides containing disulfide cyclization and/or spacer. In fact, the apparent *T*_*m*_ increased by ~ 5 °C for **(11b)** compared to the unconjugated **(11)** – again recapitulating binding data.

In aggregate, the highest thermal stability of DOTA-conjugated peptides in complex with uPAR were recorded for those peptides containing disulfide cyclization and/or C-terminal amidation – the apparent *T*_*m*_ increased by approximately 11 °C *i.e*., **(1b)** *vs.* **(3b)**,** (8b)**, and (**9b)**. For the longer DOTA-conjugated AE105 derivatives with a 6-amino acid spacer, apparent *T*_*m*_ increased by 6–14 °C upon disulfide cyclization or C-terminal amidation *i.e*., **(4b)** *vs.* **(6b)**,** (10b)**, and (**11b)**.

### In vivo PET-imaging

To obtain in vivo information on the uPAR-targeting efficacy of these new DOTA-conjugated peptides, we used them as PET probes for non-invasive imaging of uPAR expression in U87MG tumor-bearing mice. The primary aim of this study was to assess whether the disulfide cyclization of the targeting core of AE105 would improve or worsen the imaging efficiency of uPAR expression by in vivo studies. We chose four different peptides for our head-to-head comparison of their performance as imaging probes in vivo: **(1b)**, **(8b)**, **(9b)**, and **(11b)** (Table 1). With that selection of DOTA-conjugated peptides, we would be able to evaluate the impact of *i)* introducing a C-terminal amidation in AE105 [**(1b)** *vs.* **(8b)**], *ii)* introducing a disulfide constraint in the amidated AE105 [**(8b)** *vs.* **(9b)**], and finally *iii)* inserting a hydrophilic N-terminal linker between DOTA and the disulfide constrained and amidated AE105 [**(9b)** *vs.* **(11b)**].

**Figure 6:**
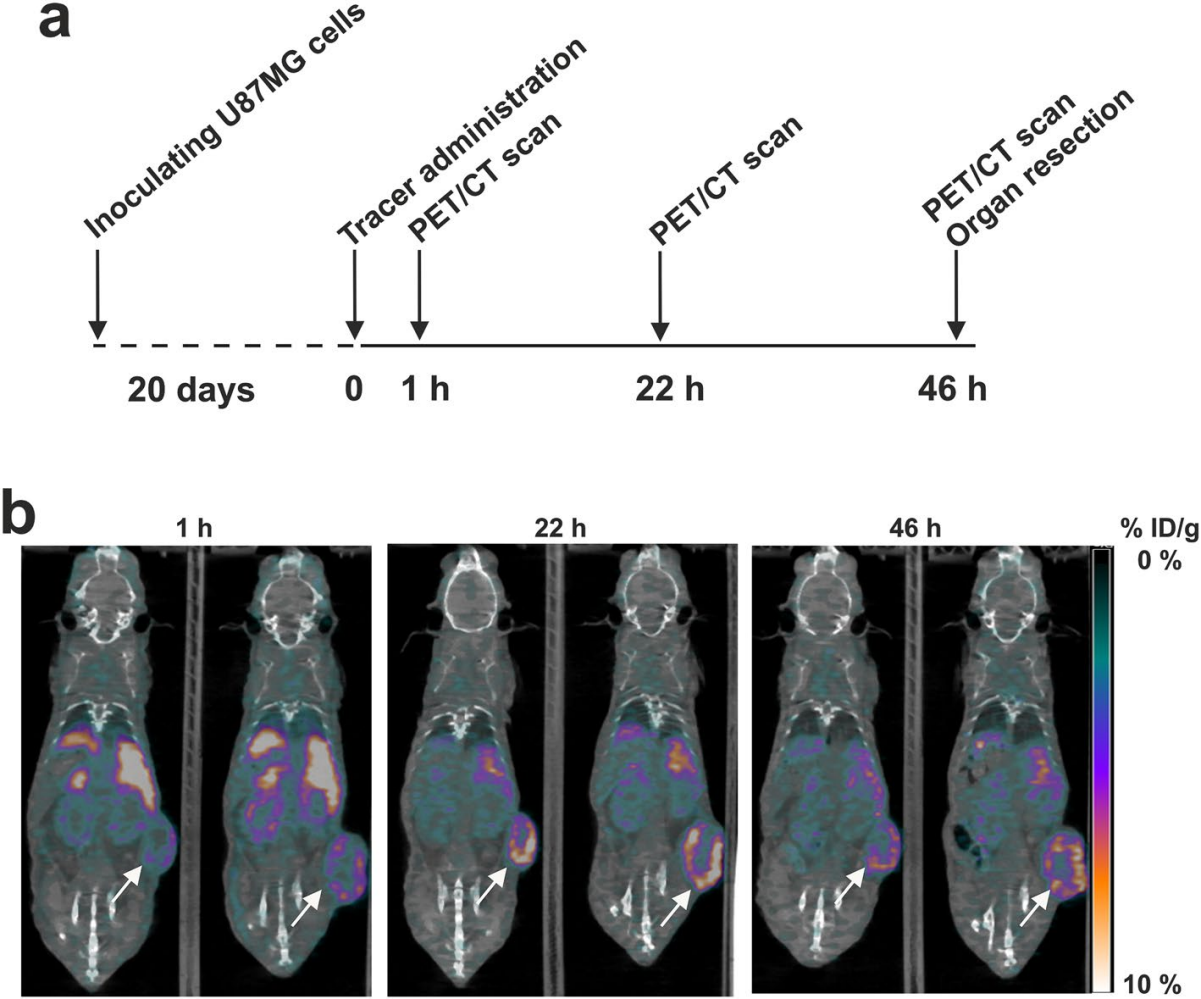
Study design and PET images of tracer uptake in U87MG tumor bearing mice. (**a**) Timeline for the in vivo experiment include 6 mice in each of the four groups. (**b**) Representative PET/CT scans performed after 1 h, 22 h, and 46 h for two mice receiving 5.4 MBq [^64^Cu]Cu-DOTA conjugated AE105 derivatives with both a [D-Cys^5^;L-Cys^9^]–disulfide and a C-terminal amidation **(9b).** The subcutaneous U87MG tumors inoculated on the right flank of female NMRI nude mice are shown by white arrows.

**Figure 7:**
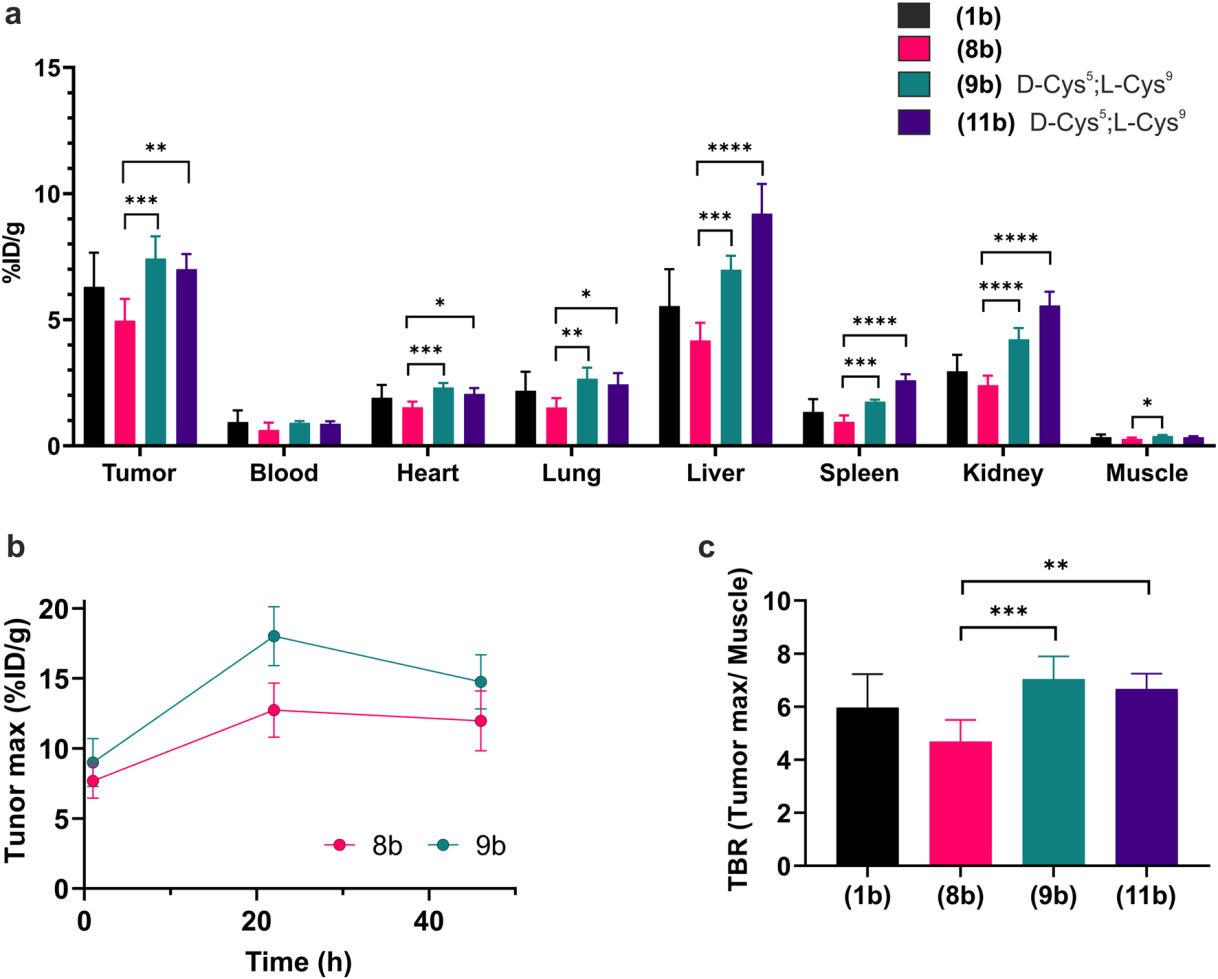
Biodistribution of [^64^Cu]Cu-DOTA–AE105 derivatives. (**a**) Organs were resected 46 h after tracer injection and the last PET scan. Their tracer content was measured by γ-counting and shown as the decay corrected tracer activity compared to the injected dose (%ID) for the stated organs with S.D. (n = 6). If a given uptake value is significantly different from that of [^64^Cu]Cu-DOTA–AE105 **(8b)** it is marked with asterisk: (*) p < 0.0332, (**) p < 0.00221, (***) p < 0.0002, (****) p < 0.0001 (Dunnett test). (**b**) Comparison of time-dependent tumor-max values for tracer accumulation of **(8b)** and its cyclized counterpart **(9b)** as measured by consecutive in vivo PET-scanning of U87MG bearing mice (mean with SD; n = 6)—the complete in vivo biodistribution is shown in Fig S6. (**c**) Tumor to muscle ratio from the ex vivo data obtained by γ-counting of resected organs (**a**).

All four DOTA-peptides were chelated with ^64^Cu and purified according to the protocols listed in Materials and Methods, which yielded preparations with radiochemical purities > 99%. Labeling at elevated temperatures did not impair their high affinity for uPAR (Fig S5). The [^64^Cu]Cu-DOTA conjugated peptides [**(1b)**, **(8b)**, **(9b)**, and **(11b)**] were immediately administered in doses of 5.4 ± 0.4 MBq by tail vein injections in U87MG tumor-bearing mice (*n* = 6 per group). The time-line for recording the non-invasive PET/CT scans is shown in Fig. 6a. Representative PET/CT images recorded at 1 h, 22 h, and 46 h after tracer administration are shown in Fig. 6b for two mice receiving [^64^Cu]Cu-DOTA conjugated **(9b)**. Compilation of time-dependent in vivo tracer uptake in tumors and selected organs by PET-CT are shown in Fig S6, while the corresponding ratios between tumor max and muscle or heart uptake are shown in Fig S7. At study termination (46 h), the decay corrected activity of resected organs were determined after γ-counting and the corresponding biodistributions are shown in Fig. 7a.

Tumor uptake values and imaging contrast recorded in this study for **(1b)** align with those reported previously for [^64^Cu]Cu-DOTA–AE105 in xenotransplanted U87MG tumors^23,58^. Amidation of AE105 **(8b)** affected neither tumor uptake nor imaging contrast as there were no significant differences in these parameters when comparing **(1b)** and **(8b)** by PET scanning (Figs S6 and S7) or ex vivo counting of resected organs (Fig. 7).

To evaluate the impact of the disulfide [D-Cys^5^;L-Cys^9^] in amidated AE105, we next compared the tracer uptake values for **(9b)** *vs.* **(8b)**. As shown in Fig S6, we observed a small but consistently higher uptake for **(9b)** *vs.* **(8b)** in all PET scans and that difference was statistically significant for tumor max at 22 h. This difference remained statistically significant both for ex vivo activity measurements in resected tumors at 46 h (Fig. 7a) and for the corresponding ratios between tumor uptake and muscle or heart uptake (Fig S7 and Fig. 7c). This beneficial effect was by and large recapitulated for the cyclic peptide with a hydrophilic spacer **(11b)**. The relatively high accumulation of **(11b)** *vs.* **(9b)** in the kidney 1 h after tracer administration (Fig S6a) is likely a consequence of its increased hydrophilicity favoring a urinary based secretion.

## Discussion

The primary objective of this study was to improve the targeting core of the uPAR specific PET-probe AE105, which is currently in phase-1 and phase-2 studies for non-invasive imaging of uPAR expression in solid cancers^31,37,38,72^. Previous preclinical research on AE105 mainly focused on exploring different positron emitting radionuclides and their macrocyclic chelators *e.g.*, [^64^Cu]-DOTA, [^64^Cu]-CB-TE2A, [^64^Cu]-CB-TE2A-PA, [^68^Ga]-NODAGA, [^68^Ga]-NOTA, and [Al^18^F]-NOTA, as reviewed^30^. These studies showed that in mice, the cross-bridged chelator [^64^Cu]-CB-TE2A-PA proved superior as reporter group with a lower nonspecific liver uptake and good spatial resolution^58^, but based on practical considerations the subsequent clinical testing were however conducted with [^64^Cu]-DOTA–AE105 and [^68^Ga]-NOTA–AE105^31,72^—reporter groups with proven efficacy in clinical nuclear medicine. Since the introduction of AE105 as an uPAR-specific PET-imaging probe in 2008^73^, no attempts have been made to further improve its affinity, which is the focus of the present study.

Informed by structure–function relationships in the uPAR•peptide interaction^54,55,57^, we now developed a cyclic derivative of AE105, which displays an increased helical propensity without compromising its high affinity for uPAR. We chose to use disulfides as the cyclizing chemistry for three reasons: First, introduction of disulfide bonds during peptide synthesis is easy and inexpensive. Second, any impact on the helix propensity by cyclization can be unambiguously determined with NMR and HDX-MS by comparing free peptides before and after reduction by DTT. Third, creating cyclic peptides with stapling chemistry would introduce a hydrophobic alkane bridge^74^, which would lower solubility and weaken the amphipathic nature of the small uPAR-targeting α-helix in AE105 (Fig. 1a). Furthermore, we chose to use DOTA as macrocyclic chelator for radionuclide tethering, as many clinical and preclinical uPAR-imaging studies already have been conducted with [^64^Cu]Cu-DOTA–AE105 as PET probe^31,32,73^. Importantly, we find that the penalty on uPAR affinity from conjugating DOTA to AE105 is prevented when the uPAR binding core of AE105 is constrained by cyclization. This suggests that the cyclic core of AE105 could potentially eliminate detrimental effects arising from putative long-range electrostatic interactions between D-Arg^5^ in AE105 and negative charges in the reporter groups. To increase solubility, we chose to use a six amino acid spacer between DOTA and AE105 (EEGsGG), but we predict that there is considerable freedom in the choice of hydrophilic spacers, particularly in the case of cyclic versions of AE105 where charge interference from D-Arg^5^ is avoided. In general, adding the hydrophilic spacer caused a penalty of two to threefold in uPAR affinity (Table 1). One possibility is that the negatively charged carboxylates are not sufficiently spaced from AE105. Circumstantial evidence from optical imaging with AE105 are aligned with that proposition. One study used an EE-linker just upstream of AE105, which yielded an IC_50_ of 132 nM^62^; another study used an EEEE-linker producing an IC_50_ of 77 nM^61^; while a third study used an EE–(OEG_2_) linker where the spacing from two oligoethylene glycol (OEG) units improved the IC_50_ to 20 nM^33^. Although the use of different fluorophores in those studies complicate direct comparisons, it is noteworthy that EE–(OEG_2_) and EEGsGG spacers both yielded a two to threefold decline in uPAR binding.

Despite the cyclic DOTA–AE105 variants **(9b)** and **(11b)** exhibit improved uPAR affinity compared to DOTA–AE105 **(1b)** (Table 1 and Fig S5), we found only minor differences in tumor max uptake values and in tumor-to-organ ratios (Figs. 7, S6, and S7). It is possible that the original high-affinity of Cu-DOTA–AE105 **(1b)** for uPAR (Table 1 and Fig S5) prevents further major improvements in imaging quality by affinity maturation. Notwithstanding this limitation, we do however find a significant albeit weak improvement in both tumor uptake and tumor-to-organ ratios for the cyclic peptides [**(9b)** and **(11b)**] when they are compared to the proper linear control **(8b)**—all these probes having C-terminal amidation (Fig. 7). In the clinic, most uPAR-targeted PET imaging platforms are presently conducted with [^68^Ga]Ga-NOTA–AE105 for logistic reasons^38–40,72^, but if the improved resolution provided by ^64^Cu is preferred, our studies suggest that [^64^Cu]Cu-CB-TE2A-PA–**(9)** would be an obvious candidate. The cross-bridged CB-TE2A-PA chelator would limit nonspecific tracer accumulation in the liver^75^ while the cyclic core of AE105 would diminish negative impacts from the chelator on uPAR affinity.

We expect that cyclic variants of AE105 combined with hydrophilic spacers will prove particularly useful in the development of new uPAR-targeted probes for fluorescence guided intraoperative imaging^30,34^. Lower sensitivity of near infrared fluorophores compared to positron emitting radionuclides calls for higher receptor occupancy and/or density in optical imaging compared to PET imaging. To achieve sufficient contrast during optical imaging much higher doses of fluorescent probe are thus needed—requiring higher probe solubility. This is generally achieved by adding several negative charged groups to the fluorophore, which in the case of AE105 is likely to lower uPAR affinity, while cyclic variants of AE105 is expected to be less sensitive.

## Materials and methods

### All methods were performed in accordance with the relevant guidelines and regulations.

#### Purified proteins and peptides

Recombinant human uPAR^1–283^ was expressed in stably transfected Drosophila melanogaster S2 cells and subsequently purified from the cell culture supernatant by affinity chromatography^75^. Human pro-uPA^1–411^ (containing a loss-of-function mutation in the active-site serine S356A) was expressed in S2 cells and purified by affinity chromatography^76^. Synthetic peptides were purchased from TAG Copenhagen A/S in a purity of > 95%.

### SPR competition assay

The IC_50_-values of antagonistic peptides on the uPAR•uPA interaction were determined with SPR using a Biacore T200™ or Biacore 3000™ instruments (Cytiva) essentially as described^4,56,77^. In brief, we immobilized high levels of pro-uPA^S356A^ (0.1 pmol pro-uPA/mm^2^) on a CM5 sensor chip via amine coupling. The experiments were conducted by injecting 2 nM uPAR in presence of a threefold dilution series of a given antagonistic peptide for 300 s with a flow rate of 50 µL/min at 20 °C. The high immobilization levels result in binding conditions that are entirely controlled by mass transport limitations (MTL). Accordingly, the observed association rates (*v*_*obs*_) are directly proportional to the concentrations of binding-active uPAR in solution. To determine the IC_50_, we also measured a standard curve of uPAR binding (a twofold dilution series of 0.06 to 2 nM uPAR, linear due to MTL). The running buffer was 10 mM HEPES, 150 mM NaCl, 3 mM EDTA, and 0.05% (v/v) surfactant P-20 at pH 7.4, and the regeneration buffer was 0.1 M acetic acid in 0.5 M NaCl.

### SPR kinetics

The real-time binding kinetics of uPAR•peptide interactions were measured with SPR using a Biacore T200™(Cytiva); essentially as described in^56,77^. In brief, uPAR was covalently immobilised on a CM5 sensor chip via amine coupling resulting in a surface density of 1157–1581 RU (~ 33–44 fmol/mm^2^). The binding kinetics for the peptides to immobilised uPAR were measured using a single cycle protocol. In this setup, peptides were injected as five serial two-fold dilutions with a contact time of 200 s. After the last analyte injection, the dissociation time was set to 1000 s. The sensor chip was regenerated with two consecutive injections of 0.1 M acetic acid in 0.5 M NaCl. All experiments were conducted at 20 °C with a flow rate of 50 µL/min in 10 mM HEPES, 150 mM NaCl, 3 mM EDTA, and 0.05% (v/v) surfactant P-20 at pH 7.4. The kinetic rate constants (*k*_*on*_ and *k*_*off*_) were determined by non-linear regression fitting of the curves to the simple bimolecular interaction model (BiacoreT200 Evaluation™ 3.0 software).

### NMR spectroscopy

Assignments and structural propensities for peptides **(4)**, **(5)**, **(6),** and **(7)** were achieved via spectra recorded on a Bruker 800 MHz spectrometer equipped with a cryogenic probe and Z-field gradient using natural isotope abundance (**(6)** peptide concentration 4.7 mM, **(4)** peptide concentration 3.0 mM, **(5)** peptide concentration 2.8 mM, **(7)** peptide concentration 2.8 mM; all in ddH2O; pH 7.0). **(6)** was also analyzed in reducing conditions (added 10 mM DTT). TOCSY, ROESY, ^15^N-HSQC, and ^13^C-HSQC spectra were acquired at 5 °C for assignment. Spectra were obtained via Bruker Topspin v4.0.7, transformed and processed using qMDD^78^ and NMRDraw (of NMRPipe^79^), respectively. All spectra were analyzed and assigned manually in CCPN Analysis v2.5^80^. For each nucleus, the SCS were obtained by subtracting the sequence corrected random coil chemical shifts from the observed shift. Random coil shifts were obtained from^81^, using predicted random coil C^α^ SCS for alanine as they are unknown for cyclohexyl-alanine.

### HDX-MS

*Continuous D-to-H labelling:* To probe the disulfide bond-induced protection of the backbone amides in **(11)**, D-to-H exchange experiments were conducted for **(10)** and **(11)** in the presence and absence of DTT. Completely deuterated equimolar solutions of **(10)** and **(11)** (10 μM) in PBS-D_2_O were prepared by lyophilization of protiated peptide solution aliquots followed by dissolution in PBS-D_2_O, pH_read_ 7.4, and incubated for 1 h at 37 °C in the presence or absence of DTT (25 mM). D-to-H exchange was initiated by tenfold dilution of the deuterated solutions of **(10)** and **(11)** into PBS-H_2_O (adjusted to pH 6.3 with phosphoric acid) at 0 °C. After 3 s, 6 s, 1, min and 10 min, isotopic exchange was quenched by acidification (2.5% (v/v) formic acid) and the sample (40 pmol of each peptide) was snap-frozen in liquid nitrogen.

*Continuous H-to-D labelling:* To probe the protection in **(10)** and **(11)** when in complex with uPAR, H-to-D exchange experiments were conducted for **(10)** and **(11)** in the presence of uPAR. Peptide **(10)** and **(11)** were mixed in PBS–H_2_O pH 7.4 to a final concentration of 10 µM for each peptide. Complexes between uPAR and the relevant antagonistic peptides were formed by adding uPAR in twofold molar excess and incubated for 15 min at room temperature (RT). Subsequently, samples were cooled on ice. Isotopic exchange was initiated by a tenfold dilution of the samples into PBS–D_2_O buffer at 0 °C, pH_read_ 7.4. For the uPAR•peptide complexes, aliquots containing 40 pmol of each peptide were withdrawn after 6 s, 60 s, 180 s, 1 h, 5 h and 24 h and quenched by acidification (2.5% (v/v) formic acid, 0 °C, pH 2.5). Subsequently, the quenched samples were snap-frozen in liquid nitrogen. Peptide samples without uPAR were also run to obtain a full deuterium control. For these samples, aliquots of 40 pmol of each peptide were withdrawn at 3, 6, 60, and 180 s (data not included). The 180 s time-point serves as our maximally labelled control. All samples were run in triplicates.

Unless indicated otherwise, chromatography and mass spectrometry equipment were from Waters Corporation, MA, USA. Peptide samples were mass-analyzed with electrospray ionization (ESI) mass spectrometry (MS) using an ESI Tri-Wave Ion Mobility mass spectrometer (Synapt G2) equipped with an HDX-Manager as described previously ^48,82^. The samples were desalted for 2 min with solvent A (0.23% aqueous formic acid) at 500 µL/min on a 1.0 mm × 5 mm MassPREP Micro Desalting Column at 0.2 °C, and eluted with a gradient flow comprised of Solvent A and Solvent B (0.23% formic acid in acetonitrile) at 0.2 °C. The gradient was 5–50% B for 3 min, 50–90% B for 1 min & 90–5% B for 3 min at a flow rate of 50 µL/min. Average masses were calculated using HX-Express2^83^.

### Nano-DSF

The thermal stability of the uPAR•peptide complexes was analyzed with Nano-DSF using a Prometheus NT.48™ (Nanotemper). uPAR (15 µM) was mixed with twofold excess of peptide (30 µM) in PBS pH 7.4 and incubated for 15 min at RT to ensure complex formation. Samples containing either 15 µM uPAR or 30 µM of each peptide were included as controls. The samples were loaded into standard capillaries and in triplicates. To unfold the proteins, the samples were exposed to an increasing temperature gradient from 20 to 95 °C with a ramping rate of 1 °C/min. The excitation power was set to 50% (Ex.280 nm) and the fluorescence emission collected at 330 nm and 350 nm. The melting temperatures of the complexes were determined in the PR.ThermControl software (Nanotemper) from the first derivative of the fluorescence ratio (350 nm/330 nm).

### Radiochemistry

^64^Cu was obtained from Hevesy Laboratory, DTU Nutech Risø as dry [^64^Cu]CuCl_2_ (1 GBq). [^64^Cu]CuCl_2_ was dissolved directly in the received vial with 125 µL TraceSelect water and left for 15 min with periodic gentle shaking. An ammonium acetate buffer containing gentisic acid was prepared by dissolving 77 mg ammonium acetate in 9 mL TraceSelect water. The pH of the buffer was adjusted to 8.4 + /- 0.1 with 2 N NaOH, followed by addition of 50 mg gentisic acid. Finally, the volume was adjusted to 10 mL with TraceSelect water and the pH was measured, the final pH should be 5.2 + /- 0.1. The DOTA-conjugated peptides **(1b)**, **(8b)**, **(9b),** and **(11b)** were labeled with ^64^Cu by adding 50 µL of the [^64^Cu]CuCl_2_ solution and 3 µL of the DOTA-peptide precursor (2 mg/mL) to a 1.5 mL Eppendorf tube containing 450 µL of the ammonium acetate buffer with gentisic acid. The mixture was heated to 80 °C with a Thermomixer for 5 min. This labeling procedure preserved the high affinity for uPAR as illustrated by SPR analysis for **(9b)** after 100% chelation with non-radioactive [^63^Cu]-DOTA (Fig S5). A sample of the crude reaction mixture was prepared and analyzed with radioHPLC (> 99% radiochemical conversion). Purification was carried out by transferring the reaction mixture to a dilution vial containing 10 mL MilliQ water. The diluted solution was trapped on a C18 light cartridge preconditioned with 5 mL EtOH and 5 mL MilliQ water. The cartridge was eluted with 0.5 mL EtOH into a vial containing 9.5 mL PBS yielding the final formulated tracer (> 65% radiochemical yield). A sample of each the tracer were prepared for radioHPLC analysis (> 99% radiochemical purity). The specific activities of the four purified [^64^Cu]Cu-DOTA peptides were 49.7 MBq/nmol **(1b)**, 51.1 MBq/nmol **(8b)**, 54.5 MBq/nmol **(9b),** and 68.6 MBq/nmol **(11b).**

### Ethics declarations

All animal experiments were performed according to the directive 2010/63/EU of the European Parliament and the European Council on the protection of animals used for scientific purposes and approved by the Danish Animal Experiments Inspectorate under an approved animal license (2021–15-0201-01041, approved December 15, 2021). All animal experiments were conducted according to ARRIVE guidelines.

### Cell line and animal model

Five weeks old female NMRI nude mice were purchased from Janvier (Le Genest-Saint-Isle, France) and housed in groups to acclimatize for a week upon arrival with light/dark period of 12/12 h and under controlled environmental conditions (temperature: 20–22 °C, relative humidity: 55%). Access to fresh water and standard pellet diet was provided ad libitum. Human glioblastoma cancer cell line U87MG (ATCC, Manassas, USA) was cultured in Dulbecco’s modified eagle medium (DMEM) supplemented with 10% fetal bovine serum and 1% penicillin–streptomycin at 37 °C and 5% CO_2_. Cells were harvested by trypsinization at a confluence of 70–90%. Mice were anesthetized (3–4% sevoflurane in 65% N_2_ and 35% O_2_) and ~ 4 × 10^6^ U87MG cells resuspended in 100 μL PBS were injected subcutaneously into the right flank. Tumors were allowed to grow for 20 days. Tumor sizes were monitored using caliper measurements (Volume = 0.5 x (Length x Width^2^)).

#### MicroPET/CT imaging

Tumor-bearing mice (tumor volumes (mean ± SD) of 221 ± 105 mm^3^) were grouped (*n* = 6 per group) and anesthetized (3–4% sevoflurane in 65% N_2_ and 35% O_2_) prior to imaging. The four different [^64^Cu]Cu–DOTA conjugated peptides were administered via tail vein injections of 5.4 ± 0.4 MBq corresponding to 118 ± 7 pmol **(1b)**, 111 ± 4 pmol **(8b)**, 92 ± 3 pmol **(9b),** and 75 ± 4 pmol **(11b)** (mean ± SD). Whole-body imaging consisting of a CT acquisition followed by a static PET acquisition was performed on an Inveon® small animal imaging system (Siemens Medical Systems, Malvern, PA, USA). PET/CT imaging was performed 1 h, 22 h, and 46 h post-injection (p.i.) of tracer with a 5 min, 10 min and 15 min PET scan, respectively. During scans the animals were placed on a heated bedding system to keep the body temperature stable. The PET acquisition was performed with an energy window of 350–650 keV and a time resolution of 6 ns, and data was corrected for dead time and decay. The CT scan consisted of 360 projections, with a tube voltage of 65 kV, a tube current of 500 μA and an exposure time of 400 ms per projection. Sinograms from PET scans were reconstructed using a 3-dimensional maximum a posteriori algorithm with correction for scatter and attenuation (CT-based). PET and CT images were co-registered and analyzed using Inveon Research Workplace software (Siemens Medical Solutions). The mean percentage of injected dose per grams (%ID/g) in different tissues was extracted by manually creating regions of interest (ROI) on fused PET/CT images.

### Biodistribution studies

After the last PET/CT scan at 46 h p.i., mice were euthanized by cervical dislocation under anesthesia, and tumor, blood, heart, lung, liver, spleen, kidney, and muscle tissue were resected. All tissues were weighted, and the radioactivity measured in a gamma counter (Wizard2, Perkin Elmer). Data was corrected for decay, tissue weight and injected amount of radioactivity.

### Statistical analysis

One-way ANOVA analysis of variance with Dunnett post hoc test was used to assess statistical significance differences between groups. A p-value < 0.033 was considered statistically significant. Prism 9.5.1 (GraphPad Software, La Jolla, CA, USA) was used for all statistical analysis.

### Supplementary Information

Supplementary Information.

## Data Availability

The datasets generated during and/or analyzed during the current study are available from the corresponding author upon reasonable request.

## Abbreviations

DOTA: Dodecane tetraacetic acid [2,2′,2′′,2′′′-(1,4,7,10-tetraazacyclododecane-1,4,7,10-tetrayl)tetraacetic acid]
ESI: Electrospray ionization
HDX-MS: Hydrogen–deuterium exchange mass spectrometry
LU: Ly-6/uPAR
MTL: Mass transport limitations
Nano-DSF: Nano-differential scanning fluorimetry
NMR: Nuclear magnetic resonance
NIR: Near-infra red
PET: Positron emission tomography
RT: Room temperature
SCSs: Secondary chemical shifts
SPR: Surface plasmon resonance
uPA: Urokinase-type plasminogen activator
uPAR: Urokinase-type plasminogen activator receptor
