# Supplementary material for: Targeted imaging of uPAR expression in vivo with cyclic AE105 variants
Source: Sci Rep. 2023 Oct 11;13:17248. doi: 10.1038/s41598-023-43934-w (PMC10567728; doi:10.1038/s41598-023-43934-w)
Supplement: Supplementary file 1 — Supplementary Information. [file 41598_2023_43934_MOESM1_ESM.pdf]

# Targeted imaging of uPAR expression *in vivo* with cyclic AE105 variants

Julie Maja Leth, Estella Anne Newcombe, Anne Louise Grønnemose, Jesper Tranekjær Jørgensen, Katrine Qvist, Anne Skovsbo Clausen, Line Bruhn Schneider Knudsen, Andreas Kjaer, Birthe Brandt Kragelund, Thomas Jørgen Dyreborg Jørgensen, Michael Ploug

## Supplementary figures S1–S7

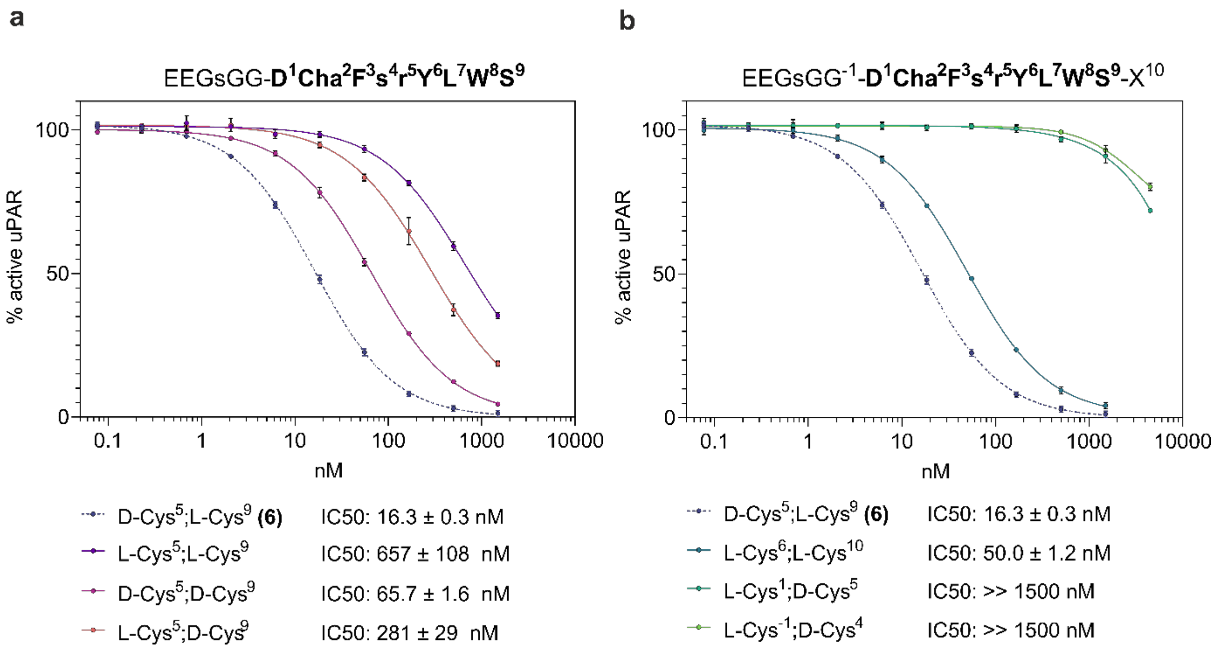


**Figure S1.** *(a)* Competition of the uPA•uPAR interaction by serial 3-fold dilutions of **(6)** and derivatives of **(6)** with different combinations in chirality of the cysteines forming the $Cys^5$;$Cys^9$ disulfide, or *(b)* derivatives with other positions of the D-$Cys^5$;L-$Cys^9$ disulfide bond.

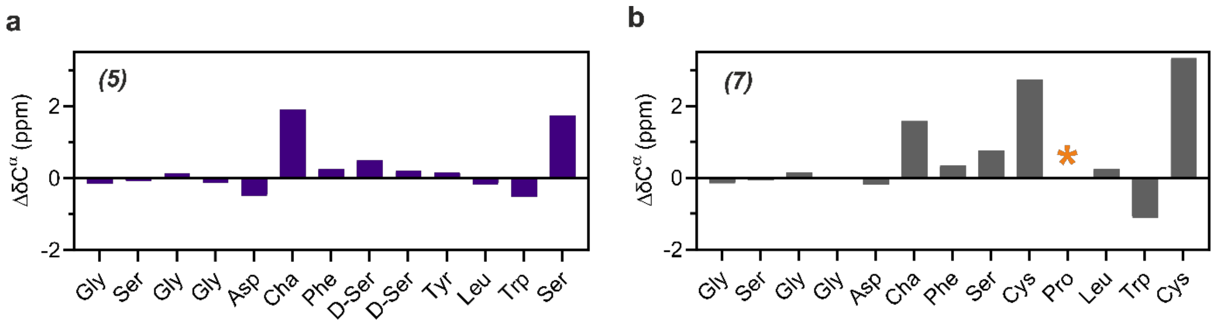


**Figure S2.** *(a)* NMR SCSs of Cα for peptide **(5)** and *(b)* peptide **(7)**. Asterisk: proline could not be assigned.

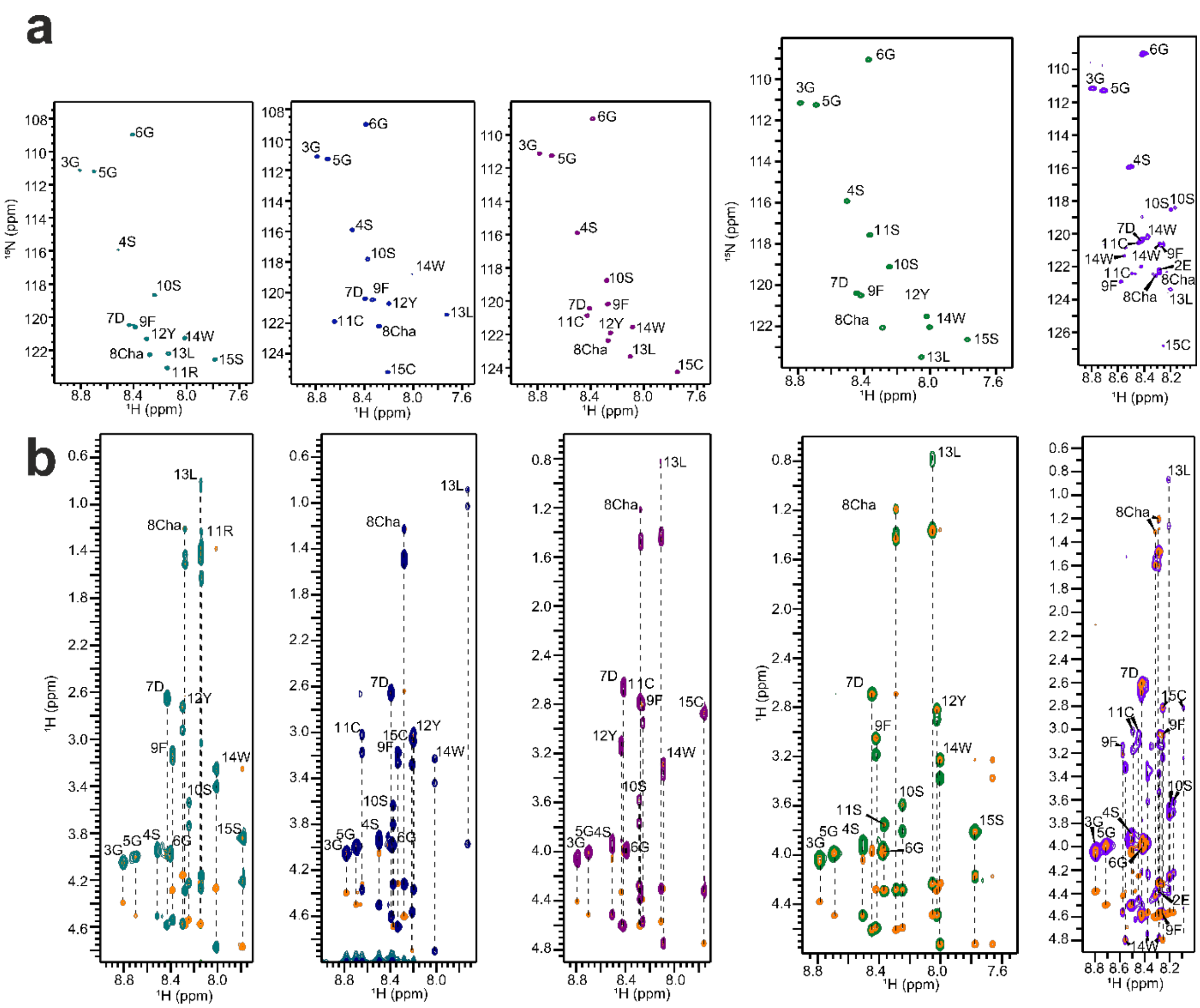

**Figure S3.** *(a)* $^{15}$N-HSQC spectra of peptide **(4)** (teal), peptide **(6)** (blue) and peptide **(6) - reduced** (purple), peptide **(5)** (green), and peptide **(7)** (light purple). *(b)* TOCSY (colored in accordance to $^{15}$N-HSQC spectra) and ROESY (orange) spectra.

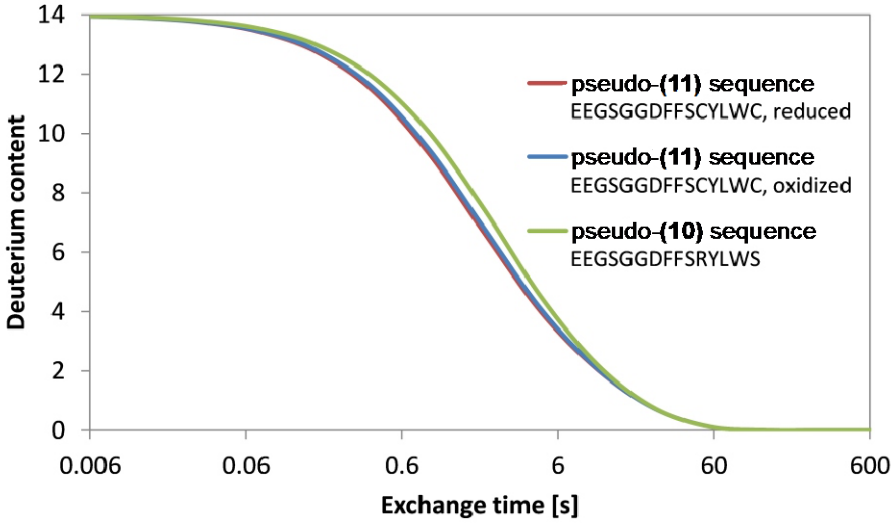


**Figure S4**. Predicted intrinsic exchange profile at pH 6.3, 0 °C as for unstructured pseudo-**(11)** (reduced & oxidized) and unstructured pseudo-**(10)** obtained from intrinsic exchange rate constants by (1).

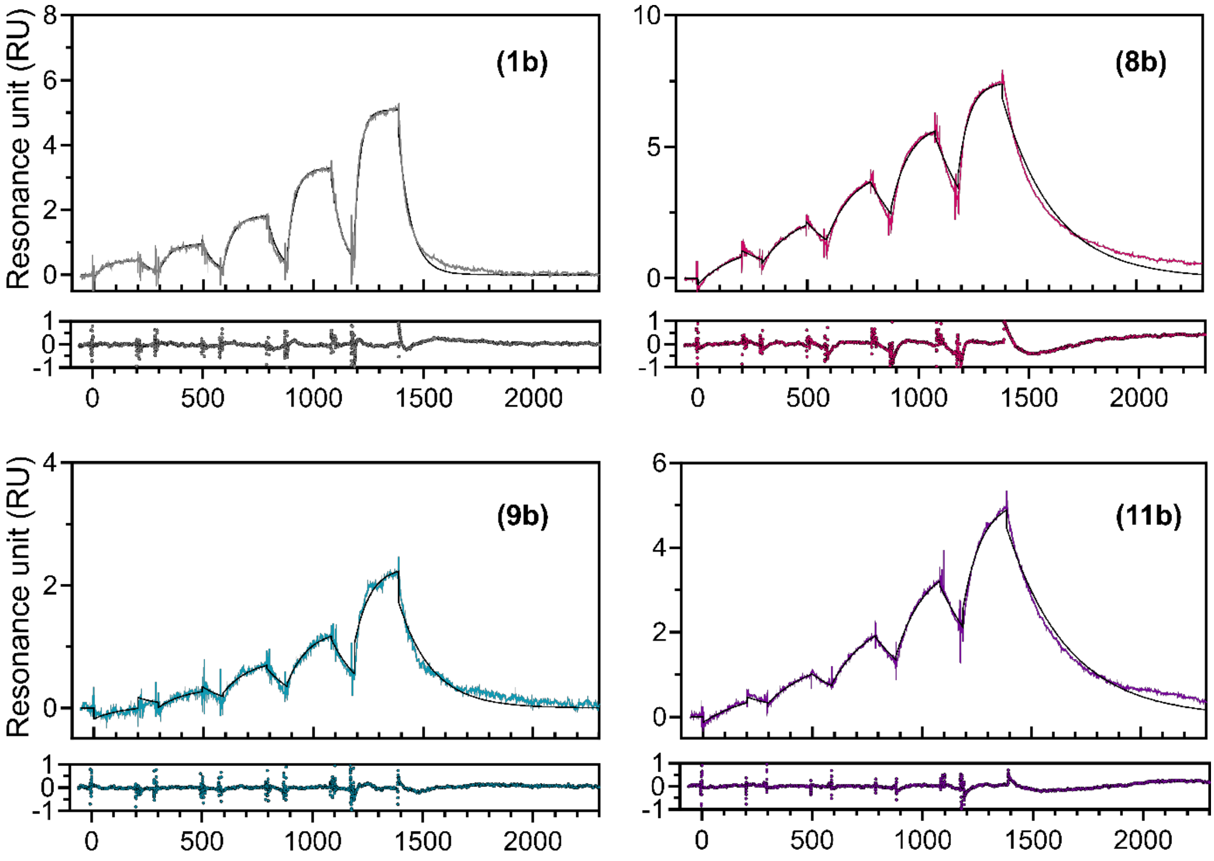


**Figure S5. uPAR affinity of Cu-labeled peptides as assessed by SPR analyses.** Real-time binding kinetics of the interactions between immobilized uPAR and [$^{63}$Cu]-DOTA labelled peptides using single-cycle injections with five 2-fold peptide dilutions: 6–100 nM **(1b)**; 3–50 nM **(8b)**; 1.6–25 nM **(9b)**; and 3–50 nM **(11b)**. Fitting to a simple 1:1 interaction model is shown by thin black line and residuals are shown beneath the sensorgram. The [$^{63}$Cu]-DOTA labelled peptides were prepared by incubating 1 mM DOTA-peptides with 1 mM $^{63}CuSO_4$ for 60 min at 70 °C in 1mM HEPES pH 7.4. Chelation of Cu was ensured by subsequent MALDI-MS analyses (2).

Fitting the data to a simple 1:1 interaction model yielded the following kinetic parameters for the individual [$^{63}$Cu]-DOTA labelled peptides:

| | Spacer | Sequence | C-term | $K_D$ (nM) | $k_{on}$ ($10^5$ $M^{-1}$ $s^{-1}$) | $k_{off}$ ($10^{-3}$ $s^{-1}$) |
|---|---|---|---|---|---|---|
| **1b** | | DChaFsrYLWS | OH | 86.5 | 2.02 ± 0.03 | 17.5 ± 0.3 |
| **8b** | | DChaFsrYLWS | $NH_2$ | 13.9 | 3.08 ± 0.01 | 4.25 ± 0.01 |
| **9b** | | DChaFs**c**YLW**C** | $NH_2$ | 22.0 | 2.99 ± 0.04 | 6.59 ± 0.02 |
| **11b** | EEGsGG | DChaFs**c**YLW**C** | $NH_2$ | 22.6 | 1.60 ± 0.01 | 3.60 ± 0.01 |

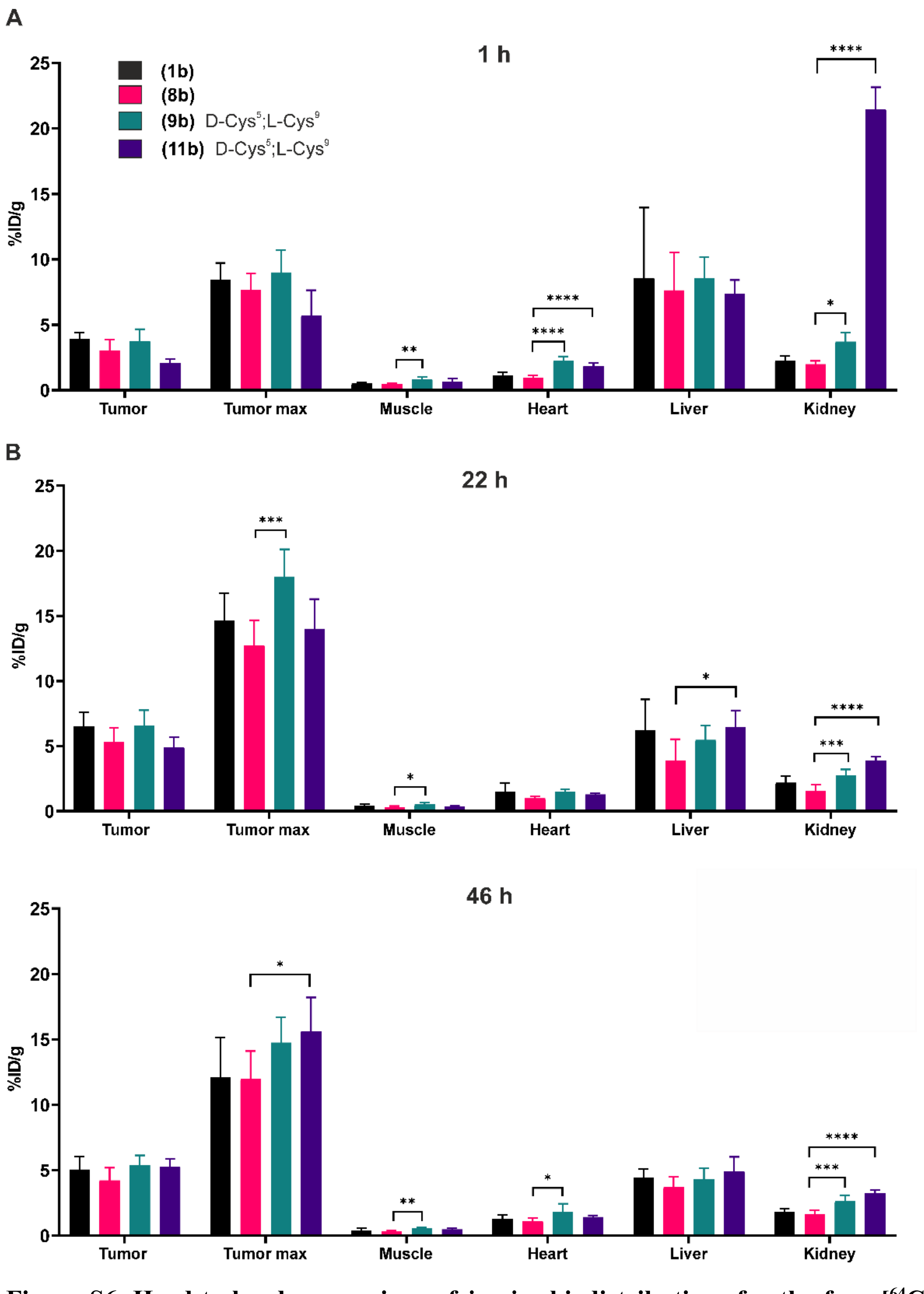


**Figure S6. Head-to-head comparison of *in vivo* biodistributions for the four [64Cu]Cu-DOTA–AE105 tracers tested by PET imaging.** PET/CT scans were recorded 1h *(a)*, 22 h *(b)*, and 46 h *(c)* after tracer administration. The bar graphs show the decay corrected tracer activity compared to the injected dose (%ID) for the stated regions of interest (mean with S.D.; n=6). If a given uptake value is significantly different from that of [64Cu]Cu-DOTA–AE105 **(8b)** it is marked with asterisk: (*) p<0.0332, (**) p<0.00221, (***) p<0.0002, (****) p<0.0001 (Dunnett test).

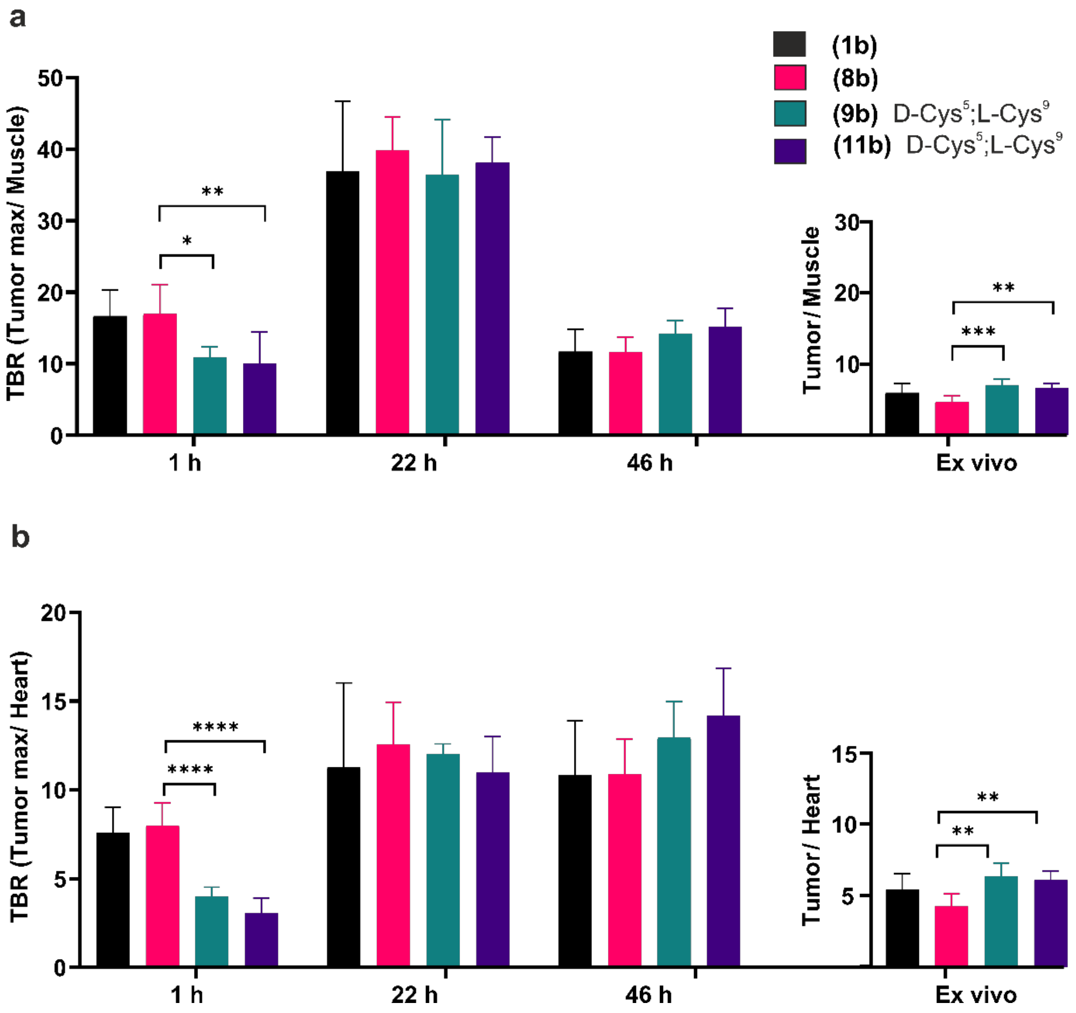


**Figure S7. Relative tumor max values for tracer accumulation of the four [$^{64}$Cu]Cu-DOTA–AE105 tracers as assessed *in vivo* by PET imaging and *ex vivo* by γ-counting.** *(a)* Time-dependent development in tumor max uptake values for **(1b)**, **(8b)**, **(9b)**, and **(11b)** relative to those measured in parallel for muscles. *(b)* Tumor max relative to heart. The *ex vivo* data shown to the right of each bar graph of *(a)* and *(b)* represents tumor to muscle ratios based on decay corrected tracer activity measured on resected organs at 46 h after tracer injection (mean with S.D.; n=6). If a given uptake value is significantly different from that of [$^{64}$Cu]Cu-DOTA–AE105 **(8b)** it is marked with asterisk: (*) $p<0.0332$, (**) $p<0.00221$, (***) $p<0.0002$ (Dunnett test).

# References


1. Bai, Y., Milne, J. S., Mayne, L., and Englander, S. W. (1993) Primary structure effects on peptide group hydrogen exchange. *Proteins* **17**, 75-86
2. Persson, M., Hosseini, M., Madsen, J., Jorgensen, T. J., Jensen, K. J., Kjaer, A., and Ploug, M. (2013) Improved PET imaging of uPAR expression using new (64)Cu-labeled cross-bridged peptide ligands: comparative in vitro and in vivo studies. *Theranostics* **3**, 618-632
